# The distribution and evolution of fungal symbioses in ancient lineages of land plants

**DOI:** 10.1007/s00572-020-00938-y

**Published:** 2020-03-04

**Authors:** William R. Rimington, Jeffrey G. Duckett, Katie J. Field, Martin I. Bidartondo, Silvia Pressel

**Affiliations:** 1 Department of Life Sciences, Imperial College London, London, SW7 2AZ UK; 2 Department of Life Sciences, Algae, Fungi and Plants Division, Natural History Museum, London, London, SW7 5BD UK; 3 Comparative Plant and Fungal Biology, Royal Botanic Gardens, Kew, Richmond, TW9 3DS UK; 4 Centre for Plant Sciences, Faculty of Biological Sciences, University of Leeds, Leeds, LS2 9JT UK

**Keywords:** Arbuscular mycorrhizas, Ericoid mycorrhizas, Mucoromycota, Hornworts, Liverworts, Lycophytes

## Abstract

An accurate understanding of the diversity and distribution of fungal symbioses in land plants is essential for mycorrhizal research. Here we update the seminal work of Wang and Qiu (Mycorrhiza 16:299-363, 2006) with a long-overdue focus on early-diverging land plant lineages, which were considerably under-represented in their survey, by examining the published literature to compile data on the status of fungal symbioses in liverworts, hornworts and lycophytes. Our survey combines data from 84 publications, including recent, post-2006, reports of Mucoromycotina associations in these lineages, to produce a list of at least 591 species with known fungal symbiosis status, 180 of which were included in Wang and Qiu (Mycorrhiza 16:299-363, 2006). Using this up-to-date compilation, we estimate that fewer than 30% of liverwort species engage in symbiosis with fungi belonging to all three mycorrhizal phyla, Mucoromycota, Basidiomycota and Ascomycota, with the last being the most widespread (17%). Fungal symbioses in hornworts (78%) and lycophytes (up to 100%) appear to be more common but involve only members of the two Mucoromycota subphyla Mucoromycotina and Glomeromycotina, with Glomeromycotina prevailing in both plant groups. Our fungal symbiosis occurrence estimates are considerably more conservative than those published previously, but they too may represent overestimates due to currently unavoidable assumptions.

**Electronic supplementary material:**

The online version of this article (10.1007/s00572-020-00938-y) contains supplementary material, which is available to authorized users.

## Introduction

Fungi colonize plants and interact with their living tissues in a variety of ways; these interactions can be detrimental (parasitic), neutral (symptomless) or beneficial (mutualistic) to the host plant. More than 85% of vascular plant species are considered to form mutually beneficial symbioses in their roots, termed mycorrhizas, with soil fungi (Brundrett and Tedersoo 2018). This percentage is only an estimate because investigating every plant species is neither practical nor currently possible given that not all species are known and ca. 2000 new vascular plants species are described each year (Pimm and Raven 2017). For the most part, fungal symbiosis occurrence rate estimates are lacking for early-diverging plant lineages as little effort has been directed towards compiling the data required to allow these estimations to be made. This also reflects an overall paucity of data available on these groups, including information on the type of interaction formed, i.e. whether the interaction is mycorrhizal or mycorrhizal-like in plants such as liverworts and hornworts that lack true roots. However, in the last decade, there has been an increased research focus on the diversity and distribution of fungal associations in liverworts, hornworts and lycophytes, largely driven by the discovery of Mucoromycotina fungi in association with these plants (Bidartondo et al. 2011; Desirò et al. 2013; Rimington et al. 2015) and the demonstration that at least some of these associations are mycorrhizal or mycorrhizal-like—i.e. those between lycophytes and Mucoromycotina (Hoysted et al. 2019); between liverworts and Glomeromycotina (Field et al. 2012), Mucoromycotina (Field et al. 2015) and Glomeromycotina and Mucoromycotina together (Field et al. 2016b); and between liverworts and Ascomycota (Kowal et al. 2018). We address this lacuna by compiling published fungal symbiosis status for these early-diverging plant lineages with the caveat that some of the reported symbioses, e.g. those in hornworts, are considered such on the basis of morphology and/or involvement of fungi known to be mycorrhizal with other plant lineages but are yet to be confirmed experimentally. A comprehensive list of which plant species enter into fungal symbioses and with which fungi not only serves as a useful resource for future studies but also provides insight into the origins and distribution of these relationships and how they evolved across plant lineages (Wang and Qiu 2006). This is particularly pertinent today as recent studies are finally providing much improved resolution on the phylogenetic relationships among the earliest-diverging bryophytes (liverworts, mosses and hornworts) and vascular plants, which have been contested for decades (e.g. Puttick et al. 2018; de Sousa et al. 2019). Within bryophytes, mosses are the only group not known to harbour symbiotic fungi in their living cells (Pressel et al. 2010). On the other hand, liverworts engage in remarkably diverse symbioses with Mucoromycotina, Glomeromycotina, Ascomycota or Basidiomycota fungi (Pressel et al. 2010; Bidartondo et al. 2011). Hornworts appear intermediate between liverworts and mosses by forming associations with Mucoromycotina and Glomeromycotina but not with members of the Dikarya (Desirò et al. 2013). Both liverworts and hornworts can also be fungus-free (non-symbiotic). Liverworts have undergone a number of gains and losses of symbiosis during their evolution; the early-diverging groups Haplomitriopsida, Marchantiopsida and Pelliidae are symbiotic with Mucoromycotina and/or Glomeromycotina (Rimington et al. 2019) while more derived lineages associate with Basidiomycota (Metzgeriidae, Jungermanniidae) and Ascomycota (Jungermanniidae) (Pressel et al. 2010). Ascomycota and Basidiomycota are both members of the subkingdom Dikarya, the latest diverging fungal lineage (Hibbett et al. 2007). Molecular analysis has indicated that the Basidiomycota symbionts of liverworts are members of the genera *Serendipita* (*Sebacina*) and *Tulasnella* (Bidartondo and Duckett 2010), while Ascomycota symbioses are formed by *Hyaloscypha* (*Pezoloma* or *Rhizoscyphus*) *ericae* (Upson et al. 2007; Fehrer et al. 2019).

Hornworts and some liverworts also form endosymbioses with cyanobacteria (*Nostoc* sp*.*) (Adams and Duggan 2008). In hornworts, these associations are ubiquitous (Renzaglia et al. 2007), while in liverworts, they occur only in two Marchantiopsida species that lack fungal symbionts, *Blasia pusilla* and *Cavicularia densa* (Rikkinen and Virtanen 2008). Associations with cyanobacteria have also been reported in some moss species; however, these are exclusively epiphytic or endophytic in the dead hyaline cells in *Sphagnum* leaves (Kostka et al. 2016; Warshan et al. 2017).

Recently, it has been shown that lycophytes also form associations with Mucoromycotina and Glomeromycotina fungi (Rimington et al. 2015), with emerging evidence of carbon-for-nutrient exchanges between these early-diverging vascular plants and their Mucoromycotina symbionts (Hoysted et al. 2019). A better understanding of fungal associations in lycophytes is important when considering the early evolution of land plant-fungus symbiosis. Lycophytes, which comprise ca. 1360 species (Hassler and Schmitt 2018), are the earliest branching lineage of vascular plants (tracheophytes) and represent the transition from non-vascular to seed plants (Kenrick and Crane 1997). They are of particular importance because putative transitional ‘pre-vascular’ plants, including Rhynie Chert fossils such as *Aglaophyton*, are all extinct (Remy et al. 1994). As such, extant lycophytes are considered the best modern analogues for the first vascular plants (Kenrick and Crane 1997).

Lists detailing the fungal symbiosis status of plants have been published for many years; for example, the first list of fungal symbiosis in liverworts was produced 70 years ago (Stahl 1949). Such lists require regular updating as the number of studies increases and so does our knowledge of the diversity of symbioses within and across plant clades. Earlier compilations usually focused on a local scale and only on certain, almost invariably vascular, plant groups (Harley and Harley 1987). It was not until 2006 that a worldwide literature survey of fungal symbioses across all land plant groups was performed (Wang and Qiu 2006). This landmark publication by Wang and Qiu (2006) captured the status of over 3000 species (143 of which were bryophytes) and, unsurprisingly, has been highly influential ever since. In the 13 years since its publication, this paper has been one of the most cited on mycorrhizas (over 1500 citations as of January 2020) and has provided important insights on the evolution of mycorrhizas; for example, evidence that arbuscular mycorrhizas (AM) are found throughout the land plant phylogeny has been used as a key argument for Glomeromycotina symbiosis being an ancestral trait of land plants (Rimington et al. 2018). However, Wang and Qiu’s survey (Wang and Qiu 2006) is now considerably outdated, especially with regard to early-diverging plant lineages. Since its publication there has been much interest in the diverse fungal symbioses of early-diverging plants (e.g. Ligrone et al. 2007; Duckett and Ligrone 2008; Bidartondo and Duckett 2010; Pressel et al. 2010; Desirò et al. 2013; Rimington et al. 2015; Rimington et al. 2018; Rimington et al. 2019) together with the discovery by Bidartondo et al. (2011) of symbioses involving Mucoromycotina fungi in liverworts, hornworts and a fern.

Fungal symbiosis occurrence rate estimates are commonly used to highlight the near-ubiquity of these relationships. For instance, few publications concerning AM fail to mention that at least 80% of plant species form these symbioses, most commonly citing the reference book ‘Mycorrhizal Symbiosis’ (Smith and Read 1997, 2008). These estimates are useful for emphasizing the importance of mycorrhizas to broad audiences and to highlight the diversity of these relationships between fungi and plants. These estimates are useful starting points for more refined estimates; recently, re-examination has shown that 80% may be an overestimation for AM symbioses, with the true value probably closer to 71% (Brundrett and Tedersoo 2018). Fungal symbiosis occurrence estimations for early-diverging plants have been more sporadic and highly variable including Glomeromycotina symbioses occurring in 60% and 100% of liverwort and hornwort species, respectively (Brundrett 2009) and 25% of bryophytes forming fungal associations, the majority of which involve Glomeromycotina (Brundrett and Tedersoo 2018). The last figure fails to take on board the fact that mosses, the most speciose group of bryophytes with ca. 12,000 species, lack fungal symbionts.

We present a new global compilation of the fungal symbiosis status of liverworts, hornworts and lycophytes. Our compilation more than triples the number of early-diverging plant species listed in Wang and Qiu (2006) and is the first to focus on early-diverging plant lineages on a global scale.

## Methods

### Literature survey

A survey of the published literature on fungal symbioses in liverworts, hornworts and lycophytes was performed. Re-examination of Wang and Qiu’s survey (Wang and Qiu 2006) revealed that some key references for these plants were missing and that fungal symbiosis status was often reported only as ‘fungal association’ without specifying the fungus involved; thus, a full search was performed**,** including studies prior to 2006. In trying to capture all available references, several keywords were used as search terms in Google Scholar. In each search, one of the following plant terms was used: ‘liverwort’, ‘hornwort’, ‘lycopod’ and ‘lycophyte’. Each plant term was combined with one of the following fungal terms: ‘fungi’, ‘fungus’, ‘Glomeromycotina’, ‘Mucoromycotina’, ‘Glomeromycota’, ‘*Glomus tenue*’ and ‘fine endophyte’. Additionally, for liverworts, which are known to form more diverse fungal symbioses than the other two lineages, the following terms were also used: ‘Ascomycota’, ‘Basidiomycota’, ‘*Rhizoscyphus*’, ‘*Pezoloma*’, ‘*Sebacina*’ and ‘*Tulasnella*’. Using these criteria, a total of 34 searches were performed. The titles and abstracts of all references returned by the searches were scrutinized to identify reports of the fungal status of any liverwort, hornwort or lycophyte species. Where the search terms returned more than 500 hits (e.g. ‘lycopod fungi’ returned 14,600 hits), only the first 500 results were investigated. Fungal symbiosis status was recorded as Glomeromycotina, Mucoromycotina, Ascomycota, Basidiomycota or non-symbiotic. Additionally, the presence of dark septate endophytes (DSE) was recorded for lycophytes. For some liverwort and hornwort species, only the presence of a ‘fungal association’ was recorded as the fungal lineage could not be assigned. As well as recording the fungal status, the identification method (microscopy and/or DNA sequencing) was noted for all species. The publications found through Google Scholar that were deemed relevant to the investigation were read and any literature found within those publications, but not returned directly by Google Scholar, was also included. This secondary search method returned exclusively microscopy studies published prior to 1990 (and dating back to 1891); thus, we are confident that all relevant molecular studies were found with our main search method. Additionally, information on the fungal symbiosis status of some liverwort species was obtained either from the liverwort flora of Paton (1999) or from our own unpublished microscopy observations (25 species; see Table S2).

### Plant nomenclature

Nomenclature for liverworts and hornworts follows the most recent floras (Söderström et al. 2016; Stotler and Crandall-Stotler 2017) and the Tropicos database (www.tropicos.org); taxonomic rankings above genus level follow Söderström et al. (2016). For lycophytes, nomenclature follows the Checklist of Ferns and Lycophytes of the World by Hassler and Schmitt (2018). When currently accepted names differ from those in the original reports, both are given in Table 1, with the latter appearing in parentheses.

**Table 1:** The fungal symbionts of early-diverging plants. Mucoro - Mucoromycotina, Glom - Glomeromycotina, Asco - Ascomycota, Basid - Basidiomycota, FA - Fungal association with unidentified fungi, NS - non-symbiotic, DSE - dark septate endophytes. Species labelled ‘Mucoro (FRE)’ were reported only as being colonized by fine root endophytes (i.e. *Glomus tenue*). A question mark after ‘Mucoro’ signifies it was not reported in the original publication but microscopy images are indicative of Mucoromycotina colonization. Checks indicate whether DNA sequencing and/or microscopy were used for fungal identification. An asterisk specifies our unpublished personal observations. In the column labelled Fungi, a hash indicates a report considered incorrect as a result of further studies. A cross signifies a likely incorrect report that is discussed in the main text. Species in bold had conflicting reports of symbiotic status. Where appropriate, the species names used in original reports are provided in parentheses. Reference numbers are listed below the table

Species	Fungi	DNA	Microscopy	Reference
**Marchantiophyta**				
Haplomitriopsida				
Haplomitriidae				
Calobryales				
Haplomitriaceae				
*Haplomitrium*				
*Haplomitrium* (*Calobryum*) *blumei*	Mucoro		✓	1–3
*Haplomitrium chilensis*	Glom^✕^	✓	✓	1
*Haplomitrium dentatum*	Mucoro	✓	✓*	4, 5
*Haplomitrium gibbsiae*	Mucoro	✓	✓	1, 2, 4–7
*Haplomitrium hookeri*	Mucoro	✓	✓	1–5, 8
*Haplomitrium intermedium*	FA		✓	1
*Haplomitrium mnioides*	Mucoro	✓	✓*	4, 5
*Haplomitrium ovalifolium*	Mucoro	✓	✓	1, 2, 6
Treubiidae				
Treubiales				
Treubiaceae				
*Treubia*				
*Treubia insignis*	FA		✓	3, 9, 10
*Treubia lacunosa*	Mucoro	✓	✓	1, 2, 4, 5, 7, 11, 12
*Treubia pygmaea*	Mucoro	✓	✓	1, 2, 4, 5, 11
*Treubia tasmanica*	Mucoro	✓	✓	2, 12
Marchantiopsida				
Blasiidae				
Blasiales				
Blasiaceae				
*Blasia*				
*Blasia pusilla*	NS (*Nostoc*)	✓	✓	1, 3, 13–16
*Cavicularia*				
*Cavicularia densa*	NS (*Nostoc*)	✓	✓	13
Marchantiidae (complex thalloid)				
Lunulariales				
Lunulariaceae				
*Lunularia*				
*Lunularia cruciata*	Glom, Mucoro	✓	✓	1, 3–5, 16–18
Marchantiales				
Aytoniaceae				
*Asterella*				
*Asterella australis*	Glom	✓	✓	1, 4, 5
*Asterella bachmannii*	Glom, Mucoro	✓	✓	1, 4, 5
*Asterella bolanderi*	Glom, Mucoro	✓		4, 5
*Asterella* (*Fimbriaria*) *blumeana*	NS		✓	3
*Asterella californica*	Mucoro	✓		4, 5
*Asterella drummondii*	NS	✓		4, 5
*Asterella grollei*	NS	✓		4, 5
*Asterella khasyana*	Glom, Mucoro	✓		4, 5
*Asterella* (*Fimbriaria*) *lindenbergiana*	NS	✓	✓	3–5
*Asterella muscicola*	Glom, Mucoro	✓	✓	1, 4, 5
*Asterella pringlei*	Mucoro	✓		4, 5
*Asterella* sp*.*	Glom, Mucoro	✓		4, 5
*Asterella* (*Fimbriaria*) sp*.*	FA		✓	3
*Asterella tenera*	Glom, Mucoro	✓	✓	1, 2, 4, 5
*Asterella wilmsii*	Glom, Mucoro	✓	✓	1, 4, 5, 19
*Cryptomitrium*				
*Cryptomitrium himalayense*	NS	✓		4, 5
*Cryptomitrium oreades*	NS	✓	✓	1, 4, 5
*Mannia*				
*Mannia angrogyna* (*Grimaldia dichotoma*)	NS		✓	1, 3
***Mannia fragrans***	NS		✓	1
“	Mucoro (FRE)		✓	20
*Mannia gracilis*	NS	✓		4, 5
*Mannia* sp	Glom	✓		4, 5
*Plagiochasma*				
***Plagiochasma eximium***	Glom		✓	1
“	NS	✓		4, 5
***Plagiochasma rupestre***	Glom, Mucoro	✓	✓	1, 4, 5, 21
“	NS		✓	3
*Plagiochasma* sp*.*	Glom, Mucoro	✓		4, 5
*Plagiochasma* sp*.*	FA		✓	3
*Reboulia*				
*Reboulia hemisphaerica*	Glom	✓	✓	1, 3–5
Cleveaceae				
*Athalamia*				
*Athalamia pinguis*	Glom	✓	✓	1, 4, 5
*Clevea*				
*Clevea* (*Athalamia*) *hyalina*	Glom	✓	✓	1, 4, 5
*Clevea spathysii* (*rousseliana*)	NS		✓	3
*Peltolepis*				
*Peltolepis quadrata* (*grandis*)	NS		✓	1, 3
*Sauteria*				
*Sauteria alpina*	NS	✓	✓	1, 3–5
Conocephalaceae				
*Conocephalum*				
*Conocephalum conicum* (*Fegatella conica*)	Glom	✓	✓	1–5, 14, 16, 22, 23
*Conocephalum japonicum*	Glom	✓		4, 5
*Conocephalum salebrosum*	Glom	✓	✓	1, 4, 5, 14
Corsiniaceae				
*Corsinia*				
***Corsinia coriandrina*(*marchantioides*)**	Glom		✓	1
“	NS	✓	✓	3–5
*Cronisia*				
*Cronisia fimbriata*	NS		✓	1
Cyathodiaceae				
*Cyathodium*				
*Cyathodium aureonitens*	NS	✓		4, 5
*Cyathodium cavernarum*	NS	✓	✓	1, 4, 5
***Cyathodium foetidissimum***	NS		✓	1
“	FA^#^		✓	3
*Cyathodium* sp.	NS	✓		4, 5
*Cyathodium tuberosum*	NS	✓		4, 5
Dumortieraceae				
*Dumortiera*				
*Dumortiera hirsuta* (*irrigua/velutina*)	Glom	✓	✓	1, 3–5
Exormothecaceae				
*Aitchisoniella*				
*Aitchisoniella himalayensis*	NS		✓	1
*Exormotheca*				
*Exormotheca holstii*	NS		✓	1, 3
*Exormotheca pustulosa*	NS		✓	1
*Stephensoniella*				
*Stephensoniella brevipedunculata*	NS		✓	1
Marchantiaceae				
*Marchantia*				
*Marchantia berteroana*	Glom	✓	✓	1, 4, 5
*Marchantia breviloba*	Glom	✓		4, 5
*Marchantia chenopoda*	Glom	✓		4, 5
*Marchantia debilis*	Glom	✓		4, 5
*Marchantia foliacea*	Glom	✓	✓	1, 2, 4, 5, 24, 25
*Marchantia geminata*	FA		✓	3
*Marchantia paleacea*	Glom	✓	✓	3–5, 26, 27
*Marchantia papillata*	Glom	✓		4, 5
*Marchantia pappeana*	Glom	✓	✓	1, 4, 5
*Marchantia pileata*	Glom	✓		4, 5
*Marchantia polymorpha* subsp. *montivagans*	Glom		✓	1
*Marchantia polymorpha* subsp. *polymorpha*	NS	✓	✓	1, 3–5
*Marchantia polymorpha* subsp. *ruderalis*	NS	✓	✓	1, 4, 5, 14
*Marchantia* (*Bucegia*) *romanica*	NS		✓	1
*Marchantia wallisii* (*grisea*)	FA		✓	3
*Preissia*				
*Preissia* (*Marchantia*) *quadrata*	Glom	✓	✓	1, 3–5, 26
Monocleaceae				
*Monoclea*				
*Monoclea forsteri*	Glom, Mucoro	✓	✓	1, 3–5
***Monoclea gottschei***	NS	✓		4, 5
“	Glom	✓	✓	1
*Monoclea* sp*.*	FA		✓	3
Monosoleniaceae				
*Monosolenium*				
*Monosolenium tenerum*	NS		✓	1
Oxymitraceae				
*Oxymitra*				
*Oxymitra cristata*	NS		✓	1
*Oxymitra incrassata*	NS	✓	✓	1, 4, 5
Ricciaceae				
*Riccia*				
*Riccia albolimbata*	NS		✓	1
*Riccia beyrichiana*	NS		✓	1
*Riccia canaliculata*	NS		✓	1
*Riccia cavernosa*	NS		✓	1
*Riccia ciliata*	NS		✓	3
*Riccia crozalsii*	NS		✓	1
*Riccia crystallina*	NS		✓	1
*Riccia fluitans*	NS		✓	1, 3, 14, 16
*Riccia glauca*	NS	✓	✓	1, 3, 14, 28
*Riccia huebeneriana*	NS		✓	1
*Riccia montana*	NS		✓	1
*Riccia nigrella*	NS		✓	1
*Riccia okahandjana*	NS		✓	1
*Riccia sorocarpa*	NS		✓	1
*Riccia stricta*	NS		✓	1
*Riccia subbifurca*	NS		✓	1
*Ricciocarpus*				
*Ricciocarpos natans*	NS		✓	1, 3
Targioniaceae				
*Targionia*				
*Targionia hypophylla*	Glom, Mucoro	✓	✓	1, 3–5
Wiesnerellaceae				
*Wiesnerella*				
***Wiesnerella denudata***	NS		✓	1
“	FA		✓	3
Neohodgsoniales				
Neohodgsoniaceae				
*Neohodgsonia*				
*Neohodgsonia mirabilis*	Glom, Mucoro	✓	✓	1, 2, 4, 5, 29
Sphaerocarpales				
Monocarpaceae				
*Monocarpus*				
*Monocarpus sphaerocarpus*	NS		✓	1
Riellaceae				
*Riella*				
*Riella americana*	NS		✓	1
*Riella helicophylla*	NS		✓	1
Sphaerocarpaceae				
*Geothallus*				
*Geothallus tuberosus*	NS		✓	1
*Sphaerocarpos*				
*Sphaerocarpos michelii*	NS		✓	1
*Sphaerocarpos texanus*	NS		✓	1
*Sphaerocarpos* sp*.*	NS		✓	3
Jungermanniopsida				
Pelliidae (simple thalloid I)				
Fossombroniales				
Calyculariaceae				
*Calycularia*				
*Calycularia crispula*	Glom, Mucoro	✓	✓*	4, 5
Allisoniaceae				
*Allisonia*				
*Allisonia cockaynei*	Glom, Mucoro	✓	✓	1, 2, 4, 5, 29
Fossombroniaceae				
*Fossombronia*				
*Fossombronia angulifolia*	Glom, Mucoro	✓	✓*	4, 5
*Fossombronia angulosa*	Glom		✓	1, 3
*Fossombronia australis*	Glom, Mucoro	✓	✓	1, 2, 4, 5
*Fossombronia caespitiformis*	Glom, Mucoro	✓	✓	1, 4, 5
*Fossombronia echinata*	Glom, Mucoro	✓	✓	1, 4, 5
*Fossombronia foveolata*	Glom, Mucoro	✓	✓	4, 5, 14
*Fossombronia husnotii*	Glom	✓	✓	4, 5
*Fossombronia hyalorhiza*	Glom, Mucoro	✓		4, 5
*Fossombronia incurva*	Glom, Mucoro	✓		4, 5
*Fossombronia indica*	Glom	✓		4, 5
*Fossombronia kashyapii*	Glom, Mucoro	✓		4, 5
*Fossombronia maritima*	Glom	✓	✓	1, 4, 5
*Fossombronia porphyrorhiza*	NS	✓		4, 5
*Fossombronia pusilla*	Glom, Mucoro	✓	✓	1, 3–5, 16
*Fossombronia reticulata*	NS	✓		4, 5
*Fossombronia* sp*.*	Glom, Mucoro	✓		4, 5
*Fossombronia wondraczekii*	Glom, Mucoro	✓	✓	1, 3–5
Petalophyllaceae				
*Petalophyllum*				
*Petalophyllum ralfsii*	Glom		✓	1
*Sewardiella*				
*Sewardiella tuberifera*	Glom, Mucoro	✓	✓	4, 5
Pallaviciniales				
Hymenophytaceae				
*Hymenophyton*				
*Hymenophyton flabellatum*	Glom	✓	✓	1, 3–5
Moerckiaceae				
*Moerckia*				
*Moerckia blyttii*	Glom, Mucoro	✓	✓	1, 3–5
*Moerckia hibernica*	NS		✓	1
*Moerckia flotoviana*	NS		✓*	
Pallaviciniaceae				
*Greeneothallus*				
*Greeneothallus gemmiparus*	Glom		✓	1
*Jensenia*				
*Jensenia connivens*	Glom	✓	✓	1, 2
*Jensenia crassifrons*	Glom	✓		4, 5
*Jensenia wallisii*	Glom		✓	1
*Pallavicinia*				
*Pallavicinia connivens*	Glom		✓	1
*Pallavicinia indica*	NS		✓	1
*Pallavicinia lyellii*	NS		✓	1
*Pallavicinia* sp*.*	FA		✓	3
***Pallavicinia xiphoides***	Glom, Mucoro	✓	✓	4, 5
“	NS^#^		✓	1
*Podomitrium*				
*Podomitrium phyllanthus*	Glom	✓	✓	1, 2, 4, 5
*Symphyogyna*				
*Symphyogyna brasiliensis*	Glom	✓	✓	1, 4, 5
*Symphyogyna brongniartii*	Glom	✓	✓	1, 4, 5
*Symphyogyna hochstetteri*	Glom, Mucoro	✓		4, 5
***Symphyogyna hymenophyllum***	Glom, Mucoro	✓	✓	1, 2, 4, 5
“	NS^#^	✓		30
*Symphyogyna podophylla*	Glom	✓		2
*Symphyogyna prolifera*	Glom	✓		2
*Symphyogyna* sp*.*	NS	✓		30
*Symphyogyna* sp*.*	FA		✓	3
*Symphyogyna subsimplex*	Glom	✓	✓	1, 2
*Symphyogyna* (*Pallavicinia*) *tenuinervis*	NS		✓	1
*Symphyogyna undulata*	Glom		✓	1
*Xenothallus*				
*Xenothallus vulcanicola*	Glom		✓	1, 25
Phyllothalliaceae				
*Phyllothallia*				
*Phyllothallia nivicola*	NS	✓	✓	1, 4, 5, 25
Pelliales				
Noterocladaceae				
*Noteroclada*				
*Noteroclada* (*Androcryphia*) *confluens*	Glom	✓	✓	1, 3–5
Pelliaceae				
*Pellia*				
***Pellia endiviifolia (fabbroniana)***	Glom	✓	✓	1, 4, 5, 14, 31
“	Mucoro (FRE)		✓	23
*Pellia epiphylla*	Glom, Mucoro	✓	✓	1, 3–5, 16
***Pellia neesiana***	NS	✓		4, 5
“	Glom		✓	1, 3
Metzgeriidae (simple thalloid II)				
Metzgeriales				
Aneuraceae				
*Aneura*				
*Aneura lobata*	Basid		✓	1
*Aneura maxima*	Basid	✓	✓	1, 3, 28
*Aneura mirabilis*	Basid	✓	✓	1, 28, 31–33
*Aneura novaguineensis*	Basid		✓	1, 32, 34
*Aneura pellioides*	NS	✓	✓	28
*Aneura pinguis*	Basid	✓	✓	1, 3, 15, 16, 28, 31, 32, 34–37
*Aneura pseudopinguis*	Basid		✓	1
*Aneura* sp.	Basid	✓		28
*Lobatiriccardia*				
*Lobatiriccardia* (*Aneura*) *alterniloba*	FA		✓	34
*Lobatiriccardia coronopus* subsp. *australis* (*Aneura lobata* subsp. *australis*)	Basid		✓	32
*Lobatiriccardia* (*Aneura*) *lobata*	Basid	✓	✓	28, 34
*Lobatiriccardia* sp.	Basid	✓		28
*Riccardia*				
*Riccardia aequicellularis*	NS		✓	34
*Riccardia aequitexta*	FA		✓	34
*Riccardia alba*	NS		✓	34
*Riccardia alcicornis*	NS		✓	34
*Riccardia asperulata*	NS		✓	34
*Riccardia australis*	FA		✓	34
*Riccardia bipinnatifda*	NS		✓	34
*Riccardia breviala*	FA		✓	34
***Riccardia chamedryfolia*(*Aneura sinuata*)**	NS	✓	✓	1, 28
“	FA		✓	3
***Riccardia cochleata***	NS		✓	1, 34
“	Basid		✓	38
*Riccardia colensoi*	NS		✓	34
*Riccardia crassa*	NS		✓	34
*Riccardia eriocaula*	NS		✓	1, 34
*Riccardia furtiva*	FA		✓	34
*Riccardia incurvata*	NS		✓	1
*Riccardia intercellula*	Basid		✓	1, 34
***Riccardia latifrons***	NS		✓	1, 33
“	Basid	✓	✓	37
*Riccardia lobulata*	NS		✓	34
*Riccardia marginata*	NS		✓	34
*Riccardia metzgeriiformis*	Basid	✓		39
*Riccardia multicorpora*	FA		✓	34
***Riccardia(Aneura)multifida***	NS	✓	✓	1, 30, 33
“	Basid	✓	✓	3, 37
*Riccardia nitida*	NS		✓	34
*Riccardia pallidevirens*	FA		✓	34
***Riccardia*(*Aneura*)*palmata***	NS		✓	1, 3, 33
“	Basid	✓	✓	37
*Riccardia papulosa*	FA		✓	34
*Riccardia pennata*	Basid		✓	1, 34, 38
*Riccardia perspicua*	FA		✓	34
*Riccardia pseudodendroceros*	NS		✓	34
*Riccardia pusilla*	FA		✓	34
*Riccardia smaragdina*	Basid	✓		35
*Riccardia* sp*.*	Basid	✓		35
*Riccardia umida*	NS		✓	34
*Riccardia wattsiana*	FA		✓	34
*Verdoornia*				
*Verdoornia succulenta*	Basid		✓	1, 25, 32
Metzgeriaceae				
*Metzgeria*				
*Metzgeria conjugata*	NS		✓	1, 33
*Metzgeria decipiens*	NS		✓	1
*Metzgeria furcata*	NS	✓	✓	1, 3, 30, 33
*Metzgeria leptoneura*	NS		✓	33
*Metzgeria pubescens*	NS		✓	1, 3, 33
*Metzgeria temperata*	NS	✓	✓	1, 30, 33
*Metzgeria violacea* (*fruticulosa*)	NS		✓	1, 33
Pleuroziales				
Pleuroziaceae				
*Pleurozia*				
*Pleurozia gigantea*	NS		✓	1
*Pleurozia purpurea*	NS	✓	✓	1, 28
Jungermanniidae (leafy)				
Jungermanniales				
Acrobolbaceae				
*Acrobolbus*				
*Acrobolbus cinerascens*	NS	✓		30
*Acrobolbus ochrophyllus*	NS		✓	25
*Acrobolbus wilsonii*	Basid^#^		✓	40
*Goebelobryum*				
*Goebelobryum unguiculatum*	NS		✓	25
*Lethocolea*				
*Lethocolea pansa*	FA		✓	25
*Saccogynidium*				
*Saccogynidium australe*	NS		✓	25
Adelanthaceae				
*Adelanthus*				
*Adelanthus bisetulus*	NS	✓		30
*Adelanthus falcatus*	FA		✓	25
*Adelanthus lindenbergianus*	NS		✓*	
*Biantheridion*				
*Biantheridion undulifolium*	NS		✓	33, 40
*Pseudomarsupidium*				
*Pseudomarsupidium* (*Adelanthus*) *decipiens*	NS		✓*	
*Syzygiella*				
***Syzygiella autumnalis***	NS		✓	40
“	FA^#^		✓	33
*Syzygiella* (*Jamesoniella*) *colorata*	NS	✓		30
*Syzygiella jacquinotii*	Asco	✓		28
*Syzygiella sonderi* (*Cryptochila grandiflora*)	NS		✓	25
*Syzygiella* (*Herzogobryum*) *teres*	NS		✓	25
*Wettsteinia*				
*Wettsteinia schusteriana*	NS	✓		30
Anastrophyllaceae				
*Anastrepta*				
*Anastrepta orcadensis*	NS		✓	40
*Anastrophyllum*				
*Anastrophyllum alpinum*	NS		✓*	
*Anastrophyllum donnianum*	NS		✓	40
*Anastrophyllum joergensenii*	NS		✓*	
*Anastrophyllum* sp*.*	NS	✓		30
*Barbilophozia*				
***Barbilophozia barbata***	Basid	✓	✓	28, 40
“	Asco^#^		✓	15
***Barbilophozia hatcheri***	Basid	✓	✓	28, 40–42
“	NS^#^	✓	✓	30, 33
*Barbilophozia* (*Lophozia*) *kunzeana*	Basid		✓	40
“	FA		✓	43, 44
*Barbilophozia lycopodioides*	Basid	✓	✓	28, 40
*Barbilophozia* (*Lophozia*) *sudetica*	Basid	✓	✓	15, 28, 40
*Crossocalyx*				
***Crossocalyx*(*Sphenolobus*)*hellerianus*(*Anastrophyllum hellerianum*)**	NS^#^		✓	33
“	Asco	✓		28
“	Basid^#^		✓	40
*Gymnocolea*				
*Gymnocolea inflata*	NS		✓	40
*Gymnocolea inflata* subsp. *acutiloba*	NS		✓	40
*Isopaches*				
*Isopaches* (*Lophozia*) *alboviridis*	FA		✓	44
*Isopaches bicrenatus* (*Lophozia bicrenata*)	Basid	✓	✓	28, 40, 43, 44
*Neoorthocaulis*				
*Neoorthocaulis* (*Barbilophozia*) *attenuatus*	Basid	✓	✓	28, 40
*Neoothocaulis* (*Barbilophozia*) *floerkei*	Basid	✓	✓	28, 40
*Orthocaulis*				
*Orthocaulis* (*Barbilophozia*) *atlanticus*	Basid		✓*	
*Schljakovia*				
*Schljakovia* (*Barbilophozia*) *kunzeana*	Basid		✓*	
*Schljakovianthus*				
*Schljakovianthus* (*Barbilophozia*) *quadrilobus* (*Lophozia quadriloba*)	Basid	✓	✓	28, 40, 44
*Sphenolobopsis*				
*Sphenolobopsis pearsonii*	NS		✓	40
*Sphenolobus*				
***Sphenolobus minutus*(*Anastrophyllum minutum*)**	NS		✓	33, 40
“	Asco^#^		✓	15
*Sphenolobus* (*Anastrophyllum*) *saxicola*	NS		✓	40
*Tetralophozia*				
*Tetralophozia setiformis*	NS	✓	✓	25, 30, 40
Antheliaceae				
*Anthelia*				
*Anthelia julacea*	NS	✓		30
*Anthelia juratzkana*	NS	✓	✓	25, 30
Balantiopsidaceae				
*Balantiopsis*				
*Balantiopsis diplophylla*	NS	✓		30
*Balantiopsis rosea*	FA		✓	25
*Isotachis*				
***Isotachis montana***	NS	✓		30
“	FA		✓	25
*Isotachis* (*Eoisotachis*) *stephanii*	FA		✓	25
Blepharostomataceae				
*Blepharostoma*				
*Blepharostoma trichophyllum*	NS		✓	33
Brevianthaceae				
*Brevianthus*				
*Brevianthus flavus*	NS	✓		30
Calypogeiaceae				
*Calypogeia*				
*Calypogeia arguta*	FA		✓	33
*Calypogeia azurea*	Asco		✓	15, 40
*Calypogeia fissa*	Asco	✓	✓	16, 28, 33, 40, 45
*Calypogeia integristipula*	Asco		✓	15, 40
***Calypogeia muelleriana***	Asco		✓	15, 16, 28, 33, 40
“	NS^#^	✓		30
*Calypogeia neesiana* (*trichomanis*)	Asco		✓	33, 40
*Calypogeia sphagnicola*	Asco		✓	25, 33
*Mizutania*				
*Mizutania riccardioides*	Asco		✓	46
Cephaloziaceae				
*Cephalozia*				
*Cephalozia ambigua*	Asco		✓*	
*Cephalozia bicuspidata*	Asco	✓	✓	16, 33, 45, 47, 48
*Cephalozia* sp*.*	NS	✓		30
*Cephalozia* sp*.*	Asco		✓	25
*Fuscocephaloziopsis*				
*Fuscocephaloziopsis* (*Pleurocladula*) *albescens*	FA		✓	49
***Fuscocephaloziopsis*(*Cephalozia*)*catenulata***	NS^#^		✓	33
“	Asco		✓*	
*Fuscocephaloziopsis* (*Cephalozia*) *connivens*	Asco	✓	✓	16, 28, 31, 33, 45, 48
*Fuscocephaloziopsis* (*Cephalozia*) *leucantha*	FA		✓	33
*Fuscocephaloziopsis* (*Cephalozia*) *loitlesbergeri*	Asco		✓	16, 33
*Fuscocephaloziopsis* (*Cephalozia*) *lunulifolia*	FA		✓	33
*Fuscocephaloziopsis* (*Cephalozia*) *macrostachya*	FA		✓	33
*Fuscocephaloziopsis* (*Schofieldia*) *monticola*	NS^#^	✓		30
*Fuscocephaloziopsis* (*Cephalozia*) *pleniceps*	FA		✓	33
*Nowellia*				
*Nowellia curvifolia*	Asco		✓	16, 33
*Odontoschisma*				
***Odontoschisma denudatum***	Asco		✓	16, 45
“	NS^#^		✓	33
*Odontoschisma elongatum*	NS		✓	33
*Odontoschisma fluitans*	NS^#^		✓	33
*Odontoschisma francisci*	FA		✓	33
*Odontoschisma macounii*	FA		✓*	
*Odontoschisma prostratum*	NS^#^	✓		30
*Odontoschisma* sp.	NS^#^	✓		30
*Odontoschisma sphagni*	FA		✓	16, 33
Cephaloziellaceae				
*Anastrophyllopsis*				
*Anastrophyllopsis subcomplicata* (*Anastrophyllum schismoides*)	NS		✓	25
*Cephaloziella*				
*Cephaloziella baumgartneri*	NS^#^		✓	33
*Cephaloziella divaricata*	Asco		✓	16, 33
*Cephaloziella exiliflora*	Asco	✓		50
*Cephaloziella hampeana*	FA		✓	33
*Cephaloziella massalongi*	NS^#^		✓	33
***Cephaloziella rubella***	FA		✓	33
“	NS^#^	✓		30
*Cephaloziella* sp*.*	Asco		✓	25
*Cephaloziella turneri*	Asco		✓*	
*Cephaloziella* (*Cephalozia*) *varians*	Asco	✓	✓	51, 52
*Nothogymnomitrion*				
*Nothogymnomitrion* (*Marsupella*) *erosum*	NS		✓	25
*Obtusifolium*				
*Obtusifolium* (*Lophozia*) *obtusum*	NS		✓	40
*Oleolophozia*				
*Oleolophozia* (*Lophozia*) *perssonii*	Basid		✓	40
*Protolophozia*				
*Protolophozia* (*Lophozia*) *crispata*	Basid	✓		28
*Protolophozia herzogiana*	FA		✓	43
Geocalycaceae				
*Geocalyx*				
***Geocalyx graveolens***	Asco	✓		28
“	Basid^#^		✓	40
“	FA		✓	33
Gymnomitriaceae				
*Gymnomitrion*				
*Gymnomitrion* (*Marsupella*) *adustum*	NS		✓	33, 40
*Gymnomitrion* (*Marsupella*) *alpinum*	NS		✓	33
*Gymnomitrion concinnatum*	NS	✓	✓	30, 33, 40
*Gymnomitrion corallioides*	NS		✓*	
*Gymnomitrion crenulatum*	NS		✓	33
*Gymnomitrion incompletum* (*cuspidatum*)	NS		✓	25
*Gymnomitrion obtusum*	NS		✓	33, 40
*Gymnomitrion* sp*.*	NS	✓		30
*Marsupella*				
*Marsupella emarginata*	NS	✓	✓	30, 33, 40
*Marsupella stableri*	NS		✓	33, 40
*Nardia*				
*Nardia breidleri*	Basid		✓	33, 40
*Nardia compressa*	NS		✓	40
*Nardia geoscyphus*	Basid	✓	✓	28, 40
***Nardia scalaris***	Basid	✓	✓	16, 28, 40, 45
“	NS^#^	✓		30
Harpanthaceae				
*Harpanthus*				
*Harpanthus flotovianus*	NS		✓	33, 40
*Harpanthus scutatus*	Basid		✓	33, 40
Herbertaceae				
*Herbertus*				
*Herbertus aduncus*	NS	✓		30
*Herbertus alpinus*	NS	✓	✓	25, 30
*Herbertus borealis*	NS		✓	33
*Triandrophyllum*				
*Triandrophyllum subtrifidum*	NS		✓	25
Hygrobiellaceae				
*Hygrobiella*				
*Hygrobiella laxifolia*	NS		✓*	
Jungermanniaceae				
*Eremonotus*				
***Eremonotus myriocarpus***	Asco	✓	✓*	28
“	Basid^#^		✓	40
*Jungermannia*				
*Jungermannia atrovirens*	NS		✓	33, 40
*Jungermannia borealis*	NS		✓	33
*Jungermannia exsertifolia*	NS		✓	33
*Jungermannia exsertifolia* subsp. *cordifolia*	NS	✓		30
*Jungermannia gracillima*	NS		✓	16, 33, 40, 45
*Jungermannia hyalina*	NS		✓	40
*Jungermannia obovata*	NS		✓	33, 40
*Jungermannia polaris*	NS		✓	40
*Jungermannia pumila*	NS		✓	33, 40
*Mesoptychia*				
*Mesoptychia* (*Leiocolea*) *badensis*	NS		✓*	
*Mesoptychia* (*Leiocolea*) *bantriensis*	NS		✓	40
*Mesoptychia* (*Leiocolea*) *heterocolpos*	NS		✓	40
*Mesoptychia* (*Leiocolea*) *rutheana*	NS		✓	40
*Mesoptychia* (*Leiocolea*) *turbinata*	NS		✓	33, 40
Lepicoleaceae				
*Lepicolea*				
*Lepicolea attenuata*	NS		✓	25
*Lepicolea scolopendra*	NS	✓	✓	25, 30
Lepidoziaceae				
*Acromastigum*				
*Acromastigum colensoanum*	FA		✓	25
*Bazzania*				
***Bazzania adnexa***	NS	✓		30
“	FA^#^		✓	25
*Bazzania denudata*	NS	✓		30
*Bazzania flaccida*	NS		✓	15
*Bazzania* sp*.*	NS	✓		30
*Bazzania tayloriana*	NS	✓		30
*Bazzania tricrenata*	NS		✓	33
***Bazzania trilobata***	Asco^#^		✓	45
“	NS	✓	✓	15, 30, 33
*Hygrolembidium*				
*Hygrolembidium australe*	Asco		✓	25
*Isolembidium*				
*Isolembidium anomalum*	Asco		✓	25
*Kurzia*				
*Kurzia pauciflora*	Asco		✓	16, 33, 45
*Kurzia* sp*.*	Asco		✓	25
*Kurzia sylvatica*	FA		✓	33
*Kurzia trichoclados*	FA		✓	33
*Lembidium*				
*Lembidium* (*Chloranthelia*) *berggrenii*	Asco		✓	25
*Lembidium nutans*	Asco		✓	25
*Lepidozia*				
***Lepidozia reptans***	Asco	✓	✓	16, 28, 33, 45
“	NS	✓		30
*Lepidozia* sp*.*	NS	✓		30
*Lepidozia* sp*.*	Asco		✓	25
*Megalembidium*				
*Megalembidium insulanum*	Asco		✓	25
*Neogrollea*				
*Neogrollea notabilis*	Asco		✓	25
*Pseudocephalozia*				
*Pseudocephalozia lepidozioides*	Asco		✓	25
*Psiloclada*				
*Psiloclada clandestina*	Asco		✓	25
*Telaranea*				
*Telaranea europaea*	Asco		✓*	
*Telaranea nematodes*	Asco		✓*	
“	FA		✓	33
*Telaranea* sp*.*	Asco		✓	25
*Tricholepidozia*				
*Tricholepidozia* (*Telaranea*) *murphyae*	Asco		✓*	
“	FA		✓	33
*Tricholepidozia* (*Telaranea*) *tetradactyla*	Asco		✓*	
*Zoopsidella*				
*Zoopsidella caledonica*	Asco		✓	25
*Zoopsis*				
*Zoopsis* sp*.*	Asco		✓	25
Lophocoleaceae				
*Chiloscyphus*				
*Chiloscyphus pallescens*	NS		✓	33, 40
*Chiloscyphus polyanthos*	NS		✓	33, 40
*Chiloscyphus* sp*.*	NS		✓	25
*Clasmatocolea*				
*Clasmatocolea* sp*.*	NS		✓	25
*Heteroscyphus*				
*Heteroscyphus billardierei*	NS	✓		30
*Heteroscyphus* sp*.*	NS		✓	25
*Leptoscyphus*				
*Leptoscyphus cuneifolius*	NS		✓	33, 40
*Leptoscyphus* sp*.*	NS		✓	25
*Lophocolea*				
*Lophocolea bidentata*	NS	✓	✓	30, 33, 40
*Lophocolea bispinosa*	NS		✓*	
*Lophocolea brookwoodiana*	NS		✓*	
*Lophocolea cuspidata*	NS		✓	33
*Lophocolea fragrans*	NS		✓*	
*Lophocolea* (*Lophozia*) *heteromorpha*	FA^#^		✓	44
***Lophocolea heterophylla***	Asco^#^		✓	15
“	NS		✓	16, 33, 40
*Lophocolea semiteres*	NS		✓*	
*Lophocolea* sp*.*	NS	✓		30
Lophoziaceae				
*Lophozia*				
*Lophozia ascendens*	FA		✓	44
*Lophozia* sp*.*	NS^#^	✓		30
*Lophozia* sp*.*	Basid		✓	25
***Lophozia ventricosa***	Basid	✓	✓	16, 28, 40, 43–45
“	NS^#^	✓		30
*Lophozia wenzelii*	Basid	✓	✓	28, 44
*Lophoziopsis*				
*Lophoziopsis* (*Lophozia*) *excisa*	Basid	✓	✓	28, 40, 43, 44, 53
*Lophoziopsis* (*Lophozia*) *latifolia*	FA		✓	44
*Lophoziopsis* (*Lophozia*) *longidens*	Basid	✓	✓	28, 40
*Lophoziopsis* (*Lophozia*) *pellucida*	FA		✓	44
*Trilophozia*				
*Trilophozia* (*Tritomaria*) *quinquedentata*	Basid		✓*	
*Tritomaria*				
***Tritomaria*(*Lophozia*)*capitata***	Basid		✓*	
“	NS		✓	40
*Tritomaria exsecta*	Basid		✓	40
*Tritomaria exsectiformis*	Basid	✓	✓	28, 40
*Tritomaria quinquidentata*	Basid	✓	✓	28, 40, 43, 44
Mastigophoraceae				
*Dendromastigophora*				
*Dendromastigophora flagellifera*	NS	✓	✓	25, 30
Myliaceae				
*Mylia*				
*Mylia anomala*	Asco		✓	33, 40
*Mylia taylorii*	NS		✓	40
Plagiochilaceae				
*Pedinophyllum*				
***Pedinophyllum interruptum***	Basid		✓	40
“	NS^#^		✓	33
“	FA		✓	43
*Plagiochila*				
*Plagiochila asplenioides*	NS	✓	✓	30, 33, 40
*Plagiochila bifaria*	NS		✓*	
*Plagiochila britannica*	NS		✓*	
*Plagiochila caduciloba*	NS	✓		30
*Plagiochila carringtonii*	NS		✓	40
*Plagiochila incurvicolla*	NS	✓		30
*Plagiochila porelloides*	NS	✓	✓	30, 33, 40
***Plagiochila punctata***	FA^#^		✓	33
“	NS		✓*	
*Plagiochila ramosissima*	NS	✓		30
*Plagiochila* sp*.*	NS	✓		30
*Plagiochila* sp*.*	NS		✓	25
*Plagiochila spinulosa*	NS		✓	33
*Plagiochila virginica*	NS	✓		30
*Plagiochilion*				
*Plagiochilion conjugatum*	NS		✓	25
Pseudolepicoleaceae				
*Archeophylla*				
*Archeophylla schusteri*	NS		✓	25
*Temnoma*				
*Temnoma quadrifidum*	NS		✓	25
Saccogynaceae				
*Saccogyna*				
*Saccogyna viticulosa*	Basid	✓	✓	28, 33, 40
Scapaniaceae				
*Diplophyllum*				
***Diplophyllum albicans***	Basid	✓	✓	15, 28, 40, 43
“	NS^#^	✓	✓	16, 30, 33
***Diplophyllum apiculatum***	Basid	✓	✓	28
“	NS^#^	✓		30
***Diplophyllum dioicum***	Basid	✓	✓	25, 28
“	NS^#^	✓		30
***Diplophyllum obtusifolium***	Basid	✓	✓	28, 40, 43
“	Asco^#^		✓	15
*Diplophyllum obtusatum*	Basid		✓*	
*Diplophyllum taxifolium*	NS	✓	✓	28, 40
*Douinia*				
*Douinia ovata*	NS	✓	✓	28, 33, 40
*Saccobasis*				
*Saccobasis* (*Tritomaria*) *polita*	Basid	✓	✓	28, 44
*Scapania*				
*Scapania aequiloba*	NS		✓	40
*Scapania aspera*	NS		✓	40
*Scapania bolanderi*	Basid	✓		54
***Scapania brevicaulis*(*degenii*)**	FA		✓	49
“	NS^#^		✓	40
***Scapania calcicola***	Basid	✓	✓	28, 40
“	NS^#^		✓	33
*Scapania compacta*	NS		✓	40
*Scapania curta* (*personnii*)	FA		✓	49
***Scapania cuspiduligera***	Basid	✓	✓	28, 40
“	NS^#^		✓	33
*Scapania glaucocephala*	FA		✓	49
*Scapania glaucocephala* var. *saxicola*	FA		✓	49
*Scapania gracilis*	NS		✓	33, 40
*Scapania gymnostomophila*	Basid		✓✓	40
“	FA		✓	49
*Scapania irrigua*	Basid	✓	✓	28, 40
*Scapania lingulata* var. *microphylla*	FA		✓	49
*Scapania nemorea*	NS	✓	✓	30, 40
*Scapania nimbosa*	NS		✓	40
*Scapania obcordata*	FA		✓	49
*Scapania obcordata* var. *paradoxa*	FA		✓	49
*Scapania ornithopodioides*	NS		✓	40
*Scapania paludicola*	NS		✓*	
*Scapania scandica*	NS		✓	33
*Scapania* sp*.*	NS	✓		30
*Scapania subaplina*	NS		✓*	
*Scapania uliginosa*	NS		✓	40
***Scapania umbrosa***	Basid	✓	✓	28, 40
“	NS^#^		✓	33
*Scapania undulata*	NS	✓	✓	28, 30, 40
*Scapania zemliae* (*invisa*)	FA		✓	49
*Schistochilopsis*				
*Schistochilopsis* (*Lophozia*) *incisa*	Basid	✓	✓	15, 28, 40
*Schistochilopsis incisa* var. *opacifolia* (*Lophozia opacifolia*)	Basid	✓	✓	28, 40
*Schistochilopsis* (*Lophozia*) *hyperarctica*	FA		✓	44
Schistochilaceae				
*Schistochila*				
*Schistochila alata*	Asco		✓	55
***Schistochila appendiculata***	Asco		✓	55
“	NS	✓		30
***Schistochila balfouriana***	Asco		✓	55
“	NS	✓		30
*Schistochila childii*	Asco		✓	55
*Schistochila glaucescens*	Asco		✓	55
*Schistochila kirkiana*	Asco		✓	55
*Schistochila lamellata*	Asco		✓	55
*Schistochila laminigera*	Asco		✓	55
*Schistochila muricata*	Asco		✓	55
*Schistochila nobilis*	Asco		✓	25, 55
*Schistochila pinnatifolia*	Asco		✓	55
*Schistochila repleta*	Asco		✓	55
*Schistochila splachnophylla*	Asco	✓	✓	45, 55
*Schistochila subimmersa*	Asco	✓	✓	45, 55
*Schistochila succulenta*	Asco		✓	45, 55
Solenostomataceae				
*Solenostoma*				
*Solenostoma* (*Jungermannia*) *orbiculata*	NS		✓	25
Southbyaceae				
*Gongylanthus*				
*Gongylanthus ericetorum*	Basid		✓	40
“	FA		✓	43
*Southbya*				
*Southbya nigrella*	Basid	✓	✓	16, 28, 40, 43
*Southbya tophacea*	Basid	✓	✓	28, 31, 40, 43
Trichocoleaceae				
*Leiomitria*				
*Leiomitra lanata*	NS	✓		30
*Trichocolea*				
*Trichocolea mollissima*	NS		✓	25
*Trichocolea rigida*	NS	✓		30
*Trichocolea tomentella*	NS	✓		30
Trichotemnomataceae				
*Trichotemnoma*				
*Trichotemnoma corrugatum*	NS		✓	25
Porellales				
Frullaniaceae				
*Frullania*				
*Frullania dilatata*	NS		✓	33
*Frullania eboracensis*	NS	✓		30
*Frullania fragilifolia*	NS		✓	33
*Frullania microphylla*	NS		✓	33
*Frullania nisquallensis*	NS	✓		30
*Frullania* sp*.*	NS		✓	25
*Frullania tamarisci*	NS		✓	33
*Frullania teneriffae*	NS		✓	33
Goebeliellaceae				
*Goebeliella*				
*Goebeliella cornigera*	NS	✓		30
Jubulaceae				
*Jubula*				
*Jubula hutchinsiae*	NS		✓	33
*Jubula hutchinsiae* subsp. *pennsylvanica*	NS	✓		30
Lejeuneaceae				
*Cheilolejeunea*				
*Cheilolejeunea* (*Leucolejeunea*) *clypeata*	NS	✓		30
*Cheilolejeunea* (*Leucolejeunea*) sp*.*	NS	✓		30
*Cololejeunea*				
*Cololejeunea calcarea*	NS		✓	33
*Cololejeunea microscopica*	NS		✓	33
*Colura*				
*Colura calyptrifolia*	NS		✓	33
*Drepanolejeunea*				
*Drepanolejeunea hamatifolia*	NS		✓	33
*Harpalejeunea*				
*Harpalejeunea ovata*	NS		✓	33
*Lejeunea*				
*Lejeunea cavifolia*	NS		✓	33
*Lejeunea lamacerina*	NS		✓	33
*Lejeunea patens*	NS		✓	33
*Lejeunea ulicina*	NS	✓	✓	30, 33
*Marchesinia*				
*Marchesinia mackaii*	NS		✓	33
*Mastigolejeunea*				
*Mastigolejeunea anguiformis*	NS	✓		30
*Myriocoleopsis*				
*Myriocoleopsis* (*Cololejeunea*) *minutissima*	NS		✓	33
Lepidolaenaceae				
*Gackstroemia*				
*Gackstroemia alpina*	NS	✓	✓	25, 30
*Lepidolaena*				
*Lepidolaena* sp*.*	NS		✓	25
*Lepidolaena taylorii*	NS	✓		30
Porellaceae				
*Lepidogyna*				
*Lepidogyna* sp*.*	NS		✓	25
*Porella*				
*Porella arboris-vitae*	NS		✓	33
*Porella cordaeana*	NS		✓	33
*Porella elegantula*	NS	✓		30
*Porella navicularis*	NS	✓		30
*Porella obtusata*	NS		✓	33
*Porella pinnata*	NS	✓	✓	30, 33
*Porella platyphylla*	NS	✓	✓	30, 33
*Porella* sp*.*	NS		✓	25
Radulaceae				
*Radula*				
*Radula aquilegia*	NS		✓	33
*Radula complanata*	NS		✓	33
*Radula lindenbergiana*	NS		✓	33
*Radula* sp*.*	NS		✓	25
Ptilidiales				
Ptilidiaceae				
*Ptilidium*				
*Ptilidium ciliare*	NS	✓	✓	25, 30
*Ptilidium* sp*.*	NS	✓		30
**Anthocerotophyta**				
Anthocerotopsida				
Anthocerotales				
Anthocerotaceae				
*Anthoceros*				
*Anthoceros agrestis*	Glom, Mucoro	✓	✓	14, 56
*Anthoceros cristatus*	Mucoro	✓	✓	57
*Anthoceros fusiformis*	Mucoro	✓		56
*Anthoceros lamellatus*	Glom, Mucoro	✓		56
*Anthoceros laminiferus*	Glom, Mucoro	✓	✓	2, 25, 56
*Anthoceros punctatus*	Glom, Mucoro	✓	✓	2, 56, 58
*Anthoceros* sp*.*	Glom, Mucoro	✓	✓	56
*Folioceros*				
*Folioceros fuciformis*	Glom	✓		56
*Folioceros* sp*.*	Glom, Mucoro	✓	✓	56
Dendrocerotales				
Dendrocerotaceae				
*Dendroceros*				
*Dendroceros crispus*	NS	✓		56
*Dendroceros granulatus*	NS		✓	25
*Dendroceros validus*	NS	✓	✓	25, 56
*Megaceros*				
*Megaceros flagellaris*	NS	✓		56
*Megaceros denticulatus*	NS		✓	25
*Megaceros leptohymenius*	Glom, Mucoro	✓		56
***Megaceros pellucidus***	Glom, Mucoro	✓		56
“	NS		✓	25
*Megaceros* sp*.*	Glom, Mucoro	✓		56
*Nothoceros*				
*Nothoceros giganteus*	NS	✓	✓	25, 56
*Nothoceros vincentianus*	Glom, Mucoro	✓		56
*Phaeomegaceros*				
*Phaeomegaceros coriaceus*	Glom, Mucoro	✓	✓	25, 56
*Phaeomegaceros hirticalyx*	Mucoro	✓	✓	56
*Phaeomegaceros* sp*.*	Glom, Mucoro	✓		56
Phymatocerotales				
Phymatocerotaceae				
*Phymatoceros*				
*Phymatoceros bulbiculosus* (*Anthoceros dichotomus*)	FA		✓	3
Notothyladales				
Notothyladaceae				
*Notothylas*				
*Notothylas javanica*	Glom	✓		56
*Notothylas orbicularis*	Glom	✓		56
*Paraphymatoceros*				
*Paraphymatoceros coriaceus*	Mucoro	✓		2
*Paraphymatoceros pearsonii*	NS	✓		56
*Paraphymatoceros* sp*.*	Mucoro	✓		2
*Phaeoceros*				
*Phaeoceros carolinianus*	Glom, Mucoro	✓	✓	2, 25, 56
*Phaeoceros dendroceroides*	Glom, Mucoro	✓		56
*Phaeoceros laevis*	Glom, Mucoro	✓	✓	2, 3, 56, 59
*Phaeoceros* sp*.*	Glom, Mucoro	✓		56
Leiosporocerotopsida				
Leiosporocerotales				
Leiosporocerotaceae				
*Leiosporoceros*				
*Leiosporoceros dussii*	NS	✓		56
**Lycopodiophyta**				
Lycopodiopsida				
Lycopodiales				
Lycopodiaceae				
*Austrolycopodium*				
*Austrolycopodium* (*Lycopodium*) *fastigiatum*	Mucoro	✓		60
*Austrolycopodium* (*Lycopodium*) *magellanicum*	NS	✓		60
*Austrolycopodium* (*Lycopodium*) *paniculatum*	DSE, Glom		✓	61
*Dendrolycopodium*				
*Dendrolycopodium dendroideum*	NS	✓		60
*Dendrolycopodium obscurum*	NS	✓		60
*Diphasiastrum*				
***Diphasiastrum*(*Lycopodium*)*alpinum***	Basid^✕^	✓	✓	62
“	Glom	✓	✓	62, 63
“	NS	✓	✓	60, 64
*Diphasiastrum complanatum*	NS		✓	65
*Diphasiastrum digitatum* (*Lycopodium digitatum/L. flabelliforme*)	Glom		✓	66, 67
*Diphasiastrum issleri*	Glom		✓	63
*Diphasiastrum* (*Lycopodium*) *thyoides*	DSE		✓	68
*Diphasiastrum* (*Lycopodium*) *tristachyum*	Glom		✓	67
*Huperzia*				
*Huperzia appressa*	NS	✓		60
***Huperzia australiana***	NS	✓		60
“	Glom		✓	69
*Huperzia lucidula*	NS	✓		60
***Huperzia*(*Lycopodium*)*selago***	NS	✓		60
“	DSE		✓	64
“	Glom		✓	63, 70
***Huperzia serrata***	NS		✓	71
“	Glom		✓	70
*Huperzia serrata* var. *longipetiolata*	NS		✓	65
*Huperzia* sp*.*	NS		✓	71
*Lateristachys*				
*Lateristachys* (*Lycopodiella*) *lateralis*	Mucoro	✓		60
*Lycopodiastrum*				
*Lycopodiastrum casuarinoides*	NS		✓	65
*Lycopodiella*				
***Lycopodiella inundata***	Mucoro	✓	✓	60, 72
“	Glom		✓	63, 73
*Lycopodium*				
***Lycopodium clavatum***	NS	✓	✓	60, 70
“	DSE		✓	64, 74
“	Glom	✓	✓	63, 75–77
“	Mucoro?		✓	76
*Lycopodium clavatum* subsp. *contiguum*	Glom	✓		77
*Lycopodium japonicum*	Glom		✓	65
*Palhinhaea*				
***Palhinhaea cernua*(*Lycopodiella cernua/Lycopodium cernuum*)**	Glom	✓	✓	60, 71, 78–80
“	NS		✓	65, 74
“	Mucoro		✓	78
*Phlegmariurus*				
*Phlegmariurus* (*Huperzia*) *affinis*	Glom	✓		77
*Phlegmariurus* (*Huperzia*) *crassus*	Glom	✓		77
*Phlegmariurus* (*Huperzia*) *hamiltonii*	Glom		✓	75
*Phlegmariurus henryi*	NS		✓	65
*Phlegmariurus hypogaeus* (*Huperzia hypogaea*)	Glom	✓	✓	77
*Phlegmariurus phlegmaria* (*Huperzia phlegmaria/Lycopodium phlegmaria*)	NS	✓		60
*Phlegmariurus phyllanthus* (*Huperzia phyllantha*)	Glom		✓	79
*Phlegmariurus squarrosus* (*Huperzia squarrosa*)	Glom		✓	74
*Phlegmariurus tetragonus* (*Huperzia tetragona*)	Glom	✓		77
*Phlegmariurus urbani* (*Huperzia urbanii*)	Glom	✓		77
*Pseudodiphasium*				
*Pseudodiphasium* (*Lycopodium*) *volubile*	NS	✓		60
*Spinulum*				
***Spinulum*(*Lycopodium*)*annotinum***	Mucoro	✓		60
“	Glom		✓	63
Isoëtales				
Isoëtaceae				
*Isoëtes*				
*Isoëtes coromandelina*	Glom		✓	81
***Isoëtes echinospora***	DSE, Glom		✓	82
“	NS		✓	63
*Isoëtes histrix*	NS		✓	63
***Isoëtes lacustris***	DSE, Glom		✓	82
“	NS		✓	63
Selaginellales				
Selaginallaceae				
*Selaginella*				
*Selaginella arbuscula*	Glom		✓	79
*Selaginella biformis*	Glom		✓	65
*Selaginella bryopteris*	Glom		✓	75
*Selaginella cataphracta*	NS		✓	74
*Selaginella chrysocaulos*	NS		✓	65
*Selaginella davidii*	Glom		✓	65, 83
*Selaginella delicatula*	Glom		✓	65
*Selaginella doederleinii*	DSE, Glom		✓	75
*Selaginella finitima*	DSE, Glom		✓	84
*Selaginella fissidentoides*	DSE, Glom		✓	74
*Selaginella frondosa*	Glom		✓	65
*Selaginella furcillifolia*	Glom		✓	71
*Selaginella helferi*	NS		✓	65
*Selaginella intermedia*	Glom		✓	71
*Selaginella involvens*	Glom		✓	65
*Selaginella kraussiana*	Glom	✓	✓	60, 63
*Selaginella mairei*	Glom		✓	85
*Selaginella martensii*	Glom		✓	84
*Selaginella minutifolia*	Glom		✓	71
*Selaginella moellendorffii*	Glom		✓	83
*Selaginella monospora*	NS		✓	65
*Selaginella obtusa*	Glom		✓	74
*Selaginella pallescens*	DSE, Glom		✓	68
*Selaginella pennata*	NS	✓		60
*Selaginella picta*	Glom		✓	65
*Selaginella plana*	Glom		✓	71
*Selaginella pulvinata*	Glom		✓	65, 85
*Selaginella remotifolia*	Glom		✓	65
*Selaginella roxburghii* var. *strigosa*	Glom		✓	71
*Selaginella sanguinolenta*	Glom		✓	65
*Selaginella selaginoides*	Glom	✓	✓	60, 63
*Selaginella* sp*.*	DSE, Glom		✓	75
*Selaginella* sp*.*	Glom		✓	80
*Selaginella stipulata*	Glom		✓	71
*Selaginella wightii*	Glom		✓	80
*Selaginella willdenowii*	NS		✓	71

1. Ligrone R et al. (2007) *Am J Bot* 94:1756–1777

2. Bidartondo MI et al. (2011) *Biol Lett* 7:574–577

3. Stahl M (1949) *Planta* 37:103–148

4. Rimington WR et al. (2018) *Proc Biol Sci* 285: 20181600

5. Rimington WR et al. (2019) *Mycorrhiza* (in the press)

6. Carafa A, Duckett JG, & Ligrone R (2003) *New Phytol* 160:185–197

7. Field KJ et al. (2015) *New Phytol* 205:743–756

8. Lilienfield F (1911) *Bulletin de l’Académie des Sciences de Cracovie Sér* B:315–339

9. Goebel K (1891) *Ann Jard Bot Buitenzorg* 9:1–11

10. Grün C (1914) *Flora* 106:331–392

11. Duckett JG, Carafa A, & Ligrone R (2006a) *Am J Bot* 93:797–813

12. Schuster RM & Scott GAM (1969) *J Hattori Bot Lab* 32:219–268

13. Rikkinen J & Virtanen V (2008) *J Exp Bot* 59:1013–1021

14. Liepina L (2012) *EEB* 10:35–40

15. Kottke I et al. (2003) *Mycol Res* 107:957–968

16. Duckett JG & Read DJ (1995) *New Phytol* 129:439–477

17. Fonseca HMAC, Berbara RLL, & Pereira ML (2006) *Mycorrhiza* 16:503–508

18. Fonseca HMAC, Azevedo A, & Pereira ML (2013) *Microsc Microanal* 19:63–64

19. Ligrone R & Duckett JG (1994) *Ann Bot* 73:577–586

20. De AB (2017) *IJARBS* 4:51–58

21. Silvani VA et al. (2012) *World J Microbiol Biotechnol* 28:3393–3397

22. Ligrone R & Lopes C (1989) *New Phytolo* 111:423–433

23. Turnau K, Ronikier M, & Unrug J (1999) *Acta Soc Bot Pol* 68:63–68

24. Russell J & Bulman S (2005) *New Phytol* 165:567–579

25. Duckett JG & Ligrone R (2008) *Flora of the Liverworts and Hornworts of New Zealand, vol. I*, eds Engel J & Glenny D (Missouri Botanical Garden Press, USA), pp. 48–56

26. Field KJ et al. (2012) *Nature Commun* 3:835

27. Humphreys CP et al. (2010) *Nature Commun* 1:103

28. Bidartondo MI & Duckett JG (2010) *Proc Biol Sci* 277:485–492

29. Field KJ et al. (2016) *ISME J* 10:1514–1526

30. Davis EC & Shaw AJ (2008) *Am J Bot* 95:914–924

31. Read DJ et al. (2000) *Philos Trans R Soc Lond B Biol Sci* 355:815–830

32. Duckett JG & Ligrone R (2008) *Can J Bot* 86:346–358

33. Pocock K & Duckett JG (1985) *New Phytol* 99:281–304

34. Brown EA & Braggins JE (1989) *J Hattori Bot Lab* 66:1–132

35. Kottke I et al. (2008) *BAAE* 9:13–23

36. Preußing M et al. (2010) *Mycorrhiza* 20:147–159

37. Krause C et al. (2011) *Fungal Biol* 115:839–851

38. Pressel S et al. (2010) *Phytotaxa* 9:238–253

39. Weiß M et al. (2011) *PLOS ONE* 6:e16793

40. Duckett JG, Russell J, & Ligrone R (2006b) *Can J Bot* 84:1075–1093

41. Zhang T et al. (2013) *Extremophiles* 17:757–765

42. Newsham KK et al. (2014) *Fungal Ecol* 11:91–99

43. Paton JA (1999) *The Liverwort Flora of the British Isles* (Brill, Leiden)

44. Schuster RM (1969) *The Hepaticae and Anthocerotae of North America, Volume 2* (Columbia University Press, Columbia)

45. Pressel S, Ligrone R, & Duckett JG (2008) *Fieldiana Botany* 47:59–72

46. Pressel S, P’ng KMY, & Duckett JG (2011) *The Bryologist* 114:38–51

47. Kowal J et al. (2018) *Ann Bot* 121:221–227

48. Kowal J et al. (2016) *Funct Ecol* 30:1014–1023

49. Schuster RM (1974) *The Hepaticae and Anthocerotae of North America, Volume 3* (Columbia University Press, Columbia)

50. Chambers SM et al. (1999) *Mycol Res* 103:286–288

51. Newsham KK (2011) *Mycorrhiza* 21:231–236

52. Upson R, Read DJ, & Newsham KK (2007) *New Phytol* 176:460–471

53. Newsham KK & Bridge PD (2010) *Mycorrhiza* 20:307–313

54. Fukasawa Y, Ando Y, & Song Z (2017) *Fungal Ecol* 30:122–131

55. Pressel S et al. (2008) *Am J Bot* 95:531–541

56. Desirò A et al. (2013) *Proc Biol Sci* 280:20130207

57. Villarreal JC, Duckett JG, & Pressel S (2017) *J Bryol* 39:226–234

58. Schüßler A (2000) *Mycorrhiza* 10:15–21

59. Ligrone R (1988) *Bot Gaz* 149:92–100

60. Rimington WR et al. (2015) *New Phytol* 205:1394–1398

61. Fernández N, Messuti MI, & Fontenla S (2008) *Am Fern J* 98:117–127

62. Horn K et al. (2013) *Am J Bot* 100:2158–2174

63. Harley JL & Harley EL (1987) *New Phytol* 105:1–102

64. Treu R et al. (1995) *Mycorrhiza* 6:21–29

65. Zhi-wei Z (2000) *Mycorrhiza* 10:145–149

66. Bruce JG (1979) *Am J Bot* 66:1138–1150

67. Berch SM & Kendrick B (1982) *Mycologia* 74:769–776

68. Zubek S et al. (2010) *Am Fern J* 100:126–136

69. Laursen GA et al. (1997) *Arct Alp Res* 29:483–491

70. Takashima Y et al. (2014) *J Sustain Agr* 9:81–88

71. Kessler M et al. (2010) *Plant Biol* 12:788–793

72. Hoystead GA et al. (2019) Plant Physiol, 10.1104/pp.19.00729

73. Fuchs B & Haselwandter K (2004) *Mycorrhiza* 14:277–281

74. Kessler M et al. (2010) *BAAE* 11:329–336

75. Muthukumar T & Prabha K (2013) *Symbiosis* 59:15–33

76. Schmid E & Oberwinkler F (1993) *New Phytol* 124:69–81

77. Winther JL & Friedman WE (2008) *New Phytol* 177:790–801

78. Duckett JG & Ligrone R (1992) *Can J Bot* 70:58–72

79. Gemma JN, Koske RE, & Flynn T (1992) *Am J Bot* 79:843–852

80. Muthuraja R et al. (2014) *Am Fern J* 104:67–102

81. Radhika KP & Rodrigues BF (2007) *Aquat Bot* 86:291–294

82. Sudová R et al. (2011) *Aquat Bot* 94:183–187

83. Zhang Y, Guo L, & Liu R (2004) *Mycorrhiza* 14:25–30

84. Lara-Pérez LA et al. (2015) *Symbiosis* 65:85–92

85. Tao L, Jianping L, & Zhiwei Z (2004) *Mycorrhiza* 14:323–327

### Estimating symbiosis occurrence rates

Fungal symbiosis occurrence rates were estimated for each of the three early diverging plant lineages: liverworts, hornworts and lycophytes. The number of species per genus or family and the total number of species per lineage were based on Söderström et al. (2016) for liverworts and hornworts and on Hassler and Schmitt (2018) for lycophytes. When making estimates for hornworts and lycophytes, if a species within a genus was colonized by a fungal lineage, then it was assumed that all members of the genus have the potential to be colonized by that fungal lineage. Underlining this assumption was the finding of fungi by our own observations on fresh specimens of the same genera. The total number of species potentially colonized in a plant lineage was divided by the total number of species in that lineage and multiplied by 100 to produce an estimate for the fungal symbiosis occurrence rate. In instances where the fungal status of a genus was unknown or reported only as ‘fungal association’, the genus was not included in the calculations and the total number of species was reduced accordingly. The same method was applied to liverworts but using the family level rather than the genus, with a few exceptions where additional considerations were included in our calculations to improve the quality of our estimates:

1. Aneuraceae—This Metzgeriidae family is the most species-rich of the simple thalloid liverworts. Colonization by Basidiomycota is common in the species-poor, early-diverging genera *Aneura*, *Lobatiriccardia* and *Verdoornia* (Rabeau et al. 2017) but less so in the largest, more derived genus *Riccardia* (Pressel et al. 2010). To avoid a considerable overestimation of symbiosis by Basidiomycota in Metzgeriidae, our calculations of fungal symbiosis occurrence rates in Aneuraceae were based on the assumption that 50% of *Riccardia* species can be colonized by Basidiomycota, i.e. the ratio of symbiotic vs. non-symbiotic *Riccardia* species found by our survey (Table 1) plus our own observations on freshly collected specimens of a range of *Riccardia* species.
2. Plagiochilaceae—This is the most speciose family in the Jungermanniales with 767 species in ten genera; however, fungal symbiosis has only been reported in the four-species genus *Pedinophyllum*. For calculations, we considered *Pedinophyllum* to be the only Plagiochilaceae genus (Feldberg et al. 2010) that can be colonized by symbiotic Basidiomycota and the rest were considered non-symbiotic. Re-enforcing this assumption is that fact that neither Schuster (1980) nor Paton (1999) mention fungi other than in *Pedinophyllum*, and we have never seen them in fresh specimens of over 50 species in the family.
3. Gymnomitriaceae—This relatively speciose family (97 species) of nine genera contains only one genus (*Nardia*) for which fungal symbiosis has been reported, and the rest are non-symbiotic; thus, for calculations, we considered *Nardia* to be the only symbiotic genus in Gymnomitriaceae. As for the Plagiochilaceae, we have never seen fungi in freshly collected specimens other than in *Nardia.* The Gymnomitriaceae predominantly grow on bare rock, a substrate ill-suited to fungal symbioses.
4. Jungermanniaceae—Fungal symbiosis has only been reported in *Eremonotus*, a single species genus (Bidartondo and Duckett 2010). All the other members of this family (37 species) that have been investigated (Paton 1999; Pocock and Duckett 1985; Schuster 1969) do not enter into fungal symbiosis, so only *Eremonotus* was considered to be symbiotic in our calculations.

Numbers of species per genus/family are given in Table S1.

### Inferring fungal symbiosis status

Fungal symbiosis status was mapped onto a representative phylogenetic diagram that contained all the plant families included in this survey with the relative positions of the plant families based on previously published phylogenies for the following plant groups: Haplomitriopsida and Marchantiopsida (Flores et al. 2017), Pelliidae and Metzgeriidae (Masuzaki et al. 2010), Jungermanniidae (Forrest et al. 2006; Shaw et al. 2015; Patzak et al. 2016), hornworts (Villarreal and Renner 2013) and lycophytes (PPG1 2016).

## Results and discussion

### Plant species numbers

The fungal symbiosis status of up to 648 liverwort, hornwort and lycophyte species, belonging to 194 genera, 82 families and 23 orders, was compiled (Table 1) by combining data from 84 publications. The number of species for each of these early-diverging plant groups and the fungal lineages that colonize them are listed in Table 2. The total value, 648 species, includes seven subspecies and 53 samples identified only to the genus level (sp.) that may represent duplicates (except when they are the only entry for that genus, e.g. *Lepidogyna* sp.). Thus, at least 591 species are included in our survey (Table 2). This represents a considerable increase on the number of early-diverging plant species, 180, included in Wang and Qiu’s survey (Wang and Qiu 2006). The hornworts and lycophytes are well represented; our survey includes members of every hornwort and lycophyte family and of most genera except for one hornwort and four lycophyte genera (Table 1). The liverworts are less well represented; this is because of their higher diversity, comprising over twenty times the number of genera found in hornworts or lycophytes. While coverage for liverworts is robust at the family level and includes 72 of the 87 families (Söderström et al. 2016), this is less so at the genus level where the fungal status of 217 of the 386 genera is currently unknown. However, early-diverging lineages are well represented at the genus level with only one Haplomitriopsida, one Marchantiopsida and five Pelliidae genera not included in Table 1. The remaining 210 genera with unknown fungal symbiosis status are members of the Metzgeriidae and Jungermanniidae. This reflects a research bias, as most studies have focused on species from known symbiotic clades (e.g. 24% of Pelliidae species and 17% of Marchantiopsida species have been investigated) while neglecting those from clades considered to be largely asymbiotic. Indeed, only 5% of Jungermanniidae species have been investigated to date, reflecting that the Lejeuneaceae, the most speciose Jungermanniidae family with ca. 2000 species, is asymbiotic (Kowal et al. 2018).

**Table 2:** The numbers of early-diverging plant species for which fungal symbiosis status has been reported. M - Mucoromycotina, G - Glomeromycotina, B - Basidiomycota, A - Ascomycota, FA - Fungal association. Where reports were contradictory (symbiotic and non-symbiotic), the symbiotic report is included. The number between parentheses represents the maximum number of different species, reflecting that some species were identified as ‘sp.’ so could represent duplicates of fully identified species

	Total	M	G	B	A	FA
Liverworts	491 (538)					
Haplomitriopsida	12	9	1	0	0	2
Haplomitriidae	8	6	1	0	0	1
Treubiidae	4	3	0	0	0	1
Marchantiopsida	88 (98)	14 (16)	33 (36)	0	0	4 (7)
Blasiidae	2	0	0	0	0	0
Marchantiidae	86 (96)	14 (16)	33 (36)	0	0	4 (7)
Jungermanniopsida	391 (428)	19 (20)	40 (41)	65 (70)	59 (64)	56 (58)
Pelliidae	48 (52)	19 (20)	40 (41)	0	0	0 (2)
Metzgeriidae	52 (56)	0	0	16 (20)	0	12
Jungermanniidae	291 (320)	0	0	49 (50)	59 (64)	44
Hornworts	27 (33)					
Anthocerotopsida	26 (32)	15 (21)	14 (19)	0	0	0
Anthocerotidae	7 (9)	6 (8)	5 (7)	0	0	0
Dendrocerotidae	12 (14)	5 (7)	4 (6)	0	0	1
Notothylatidae	7 (9)	4 (6)	5 (6)	0	0	0
Leiosporocerotopsida	1	0	0	0	0	0
Lycophytes	73 (77)					
Lycopodiopsida	73 (77)	6	53 (55)	1	0	0
Lycopodiales	35 (37)	6	22	1	0	0
Isoëtales	4	0	3	0	0	0
Selaginellales	34 (36)	0	28 (30)	0	0	0

Since the survey by Wang and Qiu was published in 2006, the use of DNA sequencing to identify plant fungal symbionts has increased dramatically. To date, the fungal status of 259 fully named early-diverging plant species has been analysed by molecular methods versus only six reported in Wang and Qiu (2006).

Our survey unveiled contradictory reports on the fungal symbiotic status (symbiotic vs. non-symbiotic) of 51 species (42 liverworts, one hornwort and eight lycophytes) probably reflecting low fungal colonization levels (Rimington et al. 2015), habitat type and/or seasonal variation in colonization (personal observations) in these species. Colonization by two fungal lineages has been reported in 51 species (35 liverworts, 11 hornworts and 5 lycophytes). We found no report of more than two fungal lineages colonizing the same plant species. All dual colonisations involve either members of Mucoromycotina and Glomeromycotina (Mucoromycota) or Ascomycota and Basidiomycota (Dikarya), with the former (45 species) being more common than the latter (5 species).

### Estimating symbiosis occurrence rates

Our estimates of fungal symbiosis occurrence rates for the different fungal lineages in liverworts, hornworts and lycophytes show that fungal symbiosis appears to be the norm in hornworts and lycophytes, but not in liverworts (Table 3). Occurrence rates were easier to estimate for hornworts and lycophytes than for liverworts, as these two groups contain less species and engage in less diverse symbioses than liverworts. We estimated that 69% of hornwort species can be colonized by Mucoromycotina fungi and 78% by Glomeromycotina (Table 3). In lycophytes, colonization by Glomeromycotina is higher than by Mucoromycotina; 99% of lycophyte species can potentially form AM while only 4% are estimated to be symbiotic with Mucoromycotina (Table 3). The fungal status of each hornwort and lycophyte genus is found in Table S1.

**Table 3:** Fungal symbiosis occurrence rate estimates

	Mucoromycotina	Glomeromycotina	Basidiomycota	Ascomycota
Liverworts	4%	5%	7%	17%
Haplomitriopsida	100%	0	0	0
Marchantiopsida	22%	38%	0	0
Jungermanniopsida				
Pelliidae	97%	99%	0	0
Metzgeriidae	0	0	44%	0
Jungermanniidae	0	0	5%	20%
Hornworts	69%	78%	0	0
Lycophytes	4%	99%	0	0

Our estimates of fungal symbiosis occurrence rates in liverworts had to be calculated at the family, rather than genus, level (except for four families, as explained previously) because this group contains many more genera (ca. 386) than hornworts and lycophytes (12 and 18 genera, respectively) and the fungal symbiosis status of less than half (169) of these genera is currently known. However, the fungal symbiotic status of most liverwort families has been reported, with that of only 15 out of 87 families remaining unassigned. These 15 families all have low species numbers: less than ten species except for one family. Thus, the fungal symbiosis status of liverworts is well represented at the family level (Table S1).

We estimated that only 4% and 5% of liverwort species are colonized by Mucoromycotina and Glomeromycotina, respectively (Table 3). Symbioses involving Basidiomycota (7%) and Ascomycota (17%) appear to be more common in liverworts but an absence of fungal symbiosis is by far the prevalent state (71%). The sum of these estimates is greater than 100% due to several liverwort species forming dual colonization with both Mucoromycotina and Glomeromycotina. Below we consider the major liverwort groups individually:

Haplomitriopsida—Up to 100% of these earliest-diverging liverworts can be colonized by Mucoromycotina fungi. There has been a single molecular report of Glomeromycotina symbiosis in *Haplomitrium chilensis* (Ligrone et al. 2007); however this report was published prior to the discovery of Mucoromycotina colonization in liverworts and has since been questioned by several molecular investigations (Bidartondo et al. 2011; Field et al. 2015; Rimington et al. 2018). Presence of Mucoromycotina and not Glomeromycotina in Haplomitriopsida liverworts also agrees with the cytology of the fungus colonizing *H. chilensis* (Ligrone et al. 2007), which we now know to be typical of Mucoromycotina and not Glomeromycotina symbioses (e.g. Field et al. 2015). We have not included in our analyses a recent study by Yamamoto et al. (2019) reporting rare Glomeromycotina associations in *Haplomitrium mnioides* from Japan, with Mucoromycotina being dominant, because the lack of anatomical details (i.e. sections of colonized axes and electron microscopy) and the limited molecular analyses presented indicate that further, more rigorous studies of this species may be required.

Marchantiopsida—These are the earliest-diverging liverworts to form Glomeromycotina symbioses; however, fungal colonization is relatively low and 22% and 38% of Marchantiopsida liverworts are estimated to be colonized by Mucoromycotina and Glomeromycotina, respectively. These results are skewed by the absence of symbionts from the most speciose Marchantiopsida family, Ricciaceae (Table S1), where both terrestrial and aquatic taxa lack symbionts. When Ricciaceae is excluded from calculations, the colonization estimates increase to 43% for Mucoromycotina and 74% for Glomeromycotina.

Pelliidae—This is the latest-diverging liverwort group to form Mucoromycotina and Glomeromycotina symbioses, and colonization is common at 97% and 99%, respectively.

Metzgeriidae—Basidiomycota colonization is estimated to occur in 44% of Metzgeriidae liverworts. If no assumption of 50% colonization in *Riccardia* species was applied to our calculations (see exception 1 in ‘Methods’), then this estimate would increase to 75%.

Jungermanniidae—Ascomycota and Basidiomycota have only been reported in the Jungermanniales and are not present in the Porellales or Ptilidiales. Our calculations suggest that 5% of Jungermanniidae species can be colonized by Basidiomycota while 20% can be colonized by Ascomycota.

Our occurrence rate estimations for Glomeromycotina colonization in early-diverging land plants disagree with those published previously by Brundrett (2009), except for lycophytes. For the latter, our results agree with 100% colonization (Brundrett 2009) (Table 3). For hornworts, our estimate of 78% is lower than the previous one of 100% (Brundrett 2009), although it confirms that colonization by Glomeromycotina in hornworts in common. The most striking discrepancy is between our finding that only 5% of liverworts likely form arbuscular mycorrhizal-like associations and the 60% estimate by Brundrett (2009). Furthermore, our results indicate that previous estimates for the formation of any type of fungal symbiosis in bryophytes have also been excessive. Wang and Qiu (2006) estimated that 46% of bryophytes enter into symbiosis with fungi, whereas Brundrett and Tedersoo (2018) put this value at 25%, while also stating that in bryophytes the majority of these relationships involve Glomeromycotina fungi. In our study, after accounting for the ca. 13,000 non-symbiotic moss species, we estimate that only 11% of bryophytes enter into a symbiosis with fungi and that the most widespread symbiosis is with Ascomycota (53%) rather than Glomeromycotina (33%). The large number of species in Lepidoziaceae, within Jungermanniidae (751 species), is principally responsible for the Ascomycota occurrence rate estimate being higher than that of the other fungal lineages combined. Even though our fungal symbiosis occurrence rates are considerably lower than previously published ones, they too may represent overestimates since our calculations are based on the assumption that all members of a plant genus (or family for liverworts) can be colonized by a fungal lineage if at least one member of the genus (or family) is colonized by that lineage. While efforts were made to prevent overestimation in four liverwort families where an absence of symbiosis is common (Aneuraceae, Gymnomitriaceae, Jungermanniaceae and Plagiochilaceae), more data are needed to determine which families are fully symbiotic and for which symbiosis is more variable.

Another important consideration in these estimations is the symbiotic status of the fungi colonizing plants. All lineages of Mucoromycotina related to the Endogonales and Glomeromycotina are considered to be mycorrhizal-like when in association with early-diverging plants (Rimington et al. 2015; Field et al. 2016a, b). This is however not the case for Ascomycota and Basidiomycota, which are far more diverse than Mucoromycotina and Glomeromycotina and regularly colonize these plants as commensals or parasites (Davis and Shaw 2008). The structures formed by Ascomycota and Basidiomycota while colonizing early-diverging plants are not necessarily diagnostic of mutualisms, and thus, it is difficult to infer mutualistic, commensal or parasitic relationships based on morphology alone (Pressel et al. 2010). Therefore, morphological observations of Basidiomycota in Metzgeriidae and Ascomycota and Basidiomycota in Jungermanniidae may not necessarily reflect mycorrhizal-like relationships. An additional complication is that, at present, *Hyaloscypha* (*Pezoloma, Rhizoscyphus*) *ericae* is the only Ascomycota species for which mutualistic nutrient exchange with liverworts has been confirmed (Kowal et al. 2018); thus, reports of colonization by Ascomycota that have not been identified as *H. ericae* using DNA sequencing may not represent mutualisms. For Basidiomycota, so far only *Tulasnella* and *Serendipita* (*Sebacina*) have been reported as genera symbiotic with liverworts (Bidartondo and Duckett 2010); however, both associations await physiological tests for exchange between partners. It follows that colonization of liverworts by mycorrhizal-like Ascomycota and Basidiomycota may have been overestimated and efforts are now required to identify molecularly the fungal symbionts of these plants as well as testing for nutrient exchange.

In contrast, Mucoromycotina occurrence rates are likely underestimates, especially for lycophytes. Traditionally, the unique structures of Glomeromycotina, in particular the arbuscules, made them easily and accurately identifiable through microscopy (Smith and Read 2008). However, the recent discovery of endosymbiotic Mucoromycotina, which cannot be distinguished from Glomeromycotina cytologically (Desirò et al. 2013; Field et al. 2016a, b) together with a report that arbuscule-forming fine root endophytes may be members of the Mucoromycotina (Orchard et al. 2017), indicates that Mucoromycotina symbionts have likely been misidentified as Glomeromycotina on a number of occasions (Field et al. 2019). It is possible, therefore, that some of the reports of Glomeromycotina symbioses in Table 1 are actually incorrect, although, at present, it is not possible to determine if and how these potential misidentifications might have influenced our occurrence rate estimations.

These caveats aside, our estimates can still be considered the best fungal symbiosis occurrence rates to date for early-diverging plants. While those for early-diverging liverworts are based on fairly comprehensive information and are unlikely to change with additional data, those for later-diverging groups are likely to improve as more data become available for these plants.

### Inferring gains and losses of symbiosis

**Fig. 1:**
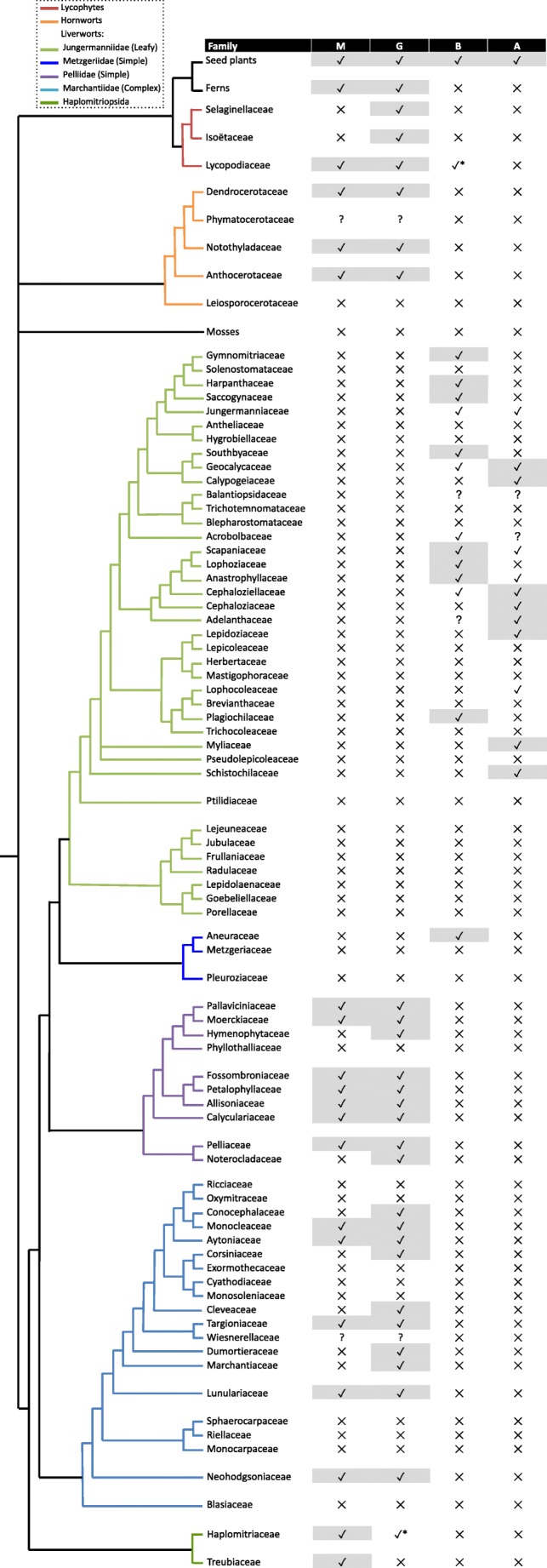
The phylogenetic position and fungal symbiosis status of early-diverging plant families. Branch lengths have no value and only show how the families are currently considered to be related. Initials in the table denote: M Mucoromycotina, G Glomeromycotina, A Ascomycota, B Basidiomycota. A check indicates presence, a cross absence, and a question mark indicates an unknown identity reported only as ‘fungal association’. Checks highlighted in grey are likely accurate reports and were used for occurrence rate estimations, whereas the mutualistic status of un-highlighted checks remains unknown (only relevant for Ascomycota and Basidiomycota symbioses in liverworts). An asterisk indicates a likely incorrect report of symbiosis

The gains and losses of fungal symbiosis during the evolutionary history of the early-diverging plant families included in Table 1 have been inferred (Fig. 1). These are discussed below:

Liverworts—The liverworts have had a more diverse history of losses and gains of symbiosis than the hornworts and lycophytes. Mucoromycotina likely formed the ancestral symbiosis with liverworts and appear to have been maintained as the sole symbionts in the Haplomitriopsida (Rimington et al. 2019). There have been losses of Mucoromycotina symbiosis in Marchantiopsida liverworts during the divergence of the Blasiales, Sphaerocarpales and Marchantiales. In the Marchantiales, symbiosis has been regained in three families, Monocleaceae, Aytoniaceae and Targioniaceae. In Pelliidae, Mucoromycotina symbiosis has been maintained in all families except four (Hymenophytaceae, Phyllothalliaceae, Petallophyllaceaeand Noterocladaceae). Conversely, Glomeromycotina symbiosis likely had a single origin in liverworts after the divergence of the Haplomitriopsida, followed by several losses in the Marchantiopsida, from Sphaerocarpales and six families of the Marchantiales, but only one loss in the Pelliidae, from the Phyllothalliaceae. After the divergence of the Pelliidae, there was a complete loss of both Mucoromycotina and Glomeromycotina symbioses in liverworts. Basidiomycota and Ascomycota symbioses appear to have been gained and lost multiple times during the evolution of the Metzgeriidae and Jungermanniidae. In the Metzgeriidae, there was a single gain of Basidiomycota symbiosis within the Aneuraceae and a subsequent loss from a large number of the later-diverging *Riccardia* species (Rabeau et al. 2017). Because the fungal symbiosis status of many Jungermanniidae families remains unresolved, it is not yet possible to accurately estimate gains and losses of Ascomycota and Basidiomycota symbioses in this subclass. Based on the better-studied families (highlighted in grey in Fig. 1), Ascomycota symbiosis appears to have evolved at least six times, with two major losses, while Basidiomycota symbiosis appears to have been gained on at least four occasions, with at least one loss. Alternatively, it is possible that Ascomycota and Basidiomycota symbioses had a single origin in the Jungermanniales followed by a large number of losses. Although this seems less likely, multiple losses of AM and rhizobia have been inferred in angiosperms, so until the fungal symbiosis status of these liverworts is fully resolved for all families, ancestral reconstruction will be of limited value to further our understanding of fungal associations in these plants.

Hornworts—Apart from some individual losses and apparent regains in certain hornwort species (Desirò et al. 2013), both Mucoromycotina and Glomeromycotina symbioses have been maintained throughout the Anthocerotopsida. Fungal symbiosis has never been recorded in the single species class Leiosporocerotopsida that contains the earliest-diverging extant hornwort *Leiosporoceros dussii*. *Leiosporoceros dussii* is notable not only for its lack of fungal symbiosis but also for its unique cyanobacterial symbiosis (Villarreal and Renzaglia 2006). With the order of divergence of the bryophytes under debate (Puttick et al. 2018), it is unknown whether Mucoromycotina and Glomeromycotina are both ancestral symbionts of all hornworts and were lost from Leiosporocerotopsida or whether these symbioses were gained in the hornworts only after Leiosporocerotopsida branched off. It also remains to be determined whether members of the Phymatocerotaceae are colonized by Mucoromycotina, Glomeromycotina or both fungi since the only record for this family is a report of ‘a fungal association’ (Stahl 1949); however, the regular colonization of the other Anthocerotopsida families by Mucoromycotina and Glomeromycotina suggests this family is also colonized by both fungal lineages.

Lycophytes—Phylogenetic inference (Fig. 1) and fossil evidence (Strullu-Derrien et al. 2014) both support that the ancestor of all vascular plants entered into symbiosis with Mucoromycotina and Glomeromycotina. Within the lycophytes, there have only been losses of symbiosis and no subsequent gains. The loss of Mucoromycotina symbiosis appears to have occurred on a larger scale than that of Glomeromycotina symbiosis, with a major loss after the divergence of the Lycopodiaceae which resulted in Isoëtaceae and Selaginellaceae apparently being colonized only by Glomeromycotina. It should be noted however that no fungal molecular data have been generated from Isoëtaceae and all microscopy reports predate the discovery of Mucoromycotina in lycophytes; therefore a symbiosis with Mucoromycotina cannot be ruled out. Additionally, only three of the 688 Selaginellaceae species have been analysed molecularly (Rimington et al. 2015); therefore, this family may also enter into symbiosis with Mucoromycotina as well as Glomeromycotina. There have also been losses of Mucoromycotina symbiosis within the Lycopodiaceae and the subfamily Huperzoideae is only colonized by Glomeromycotina. Within the subfamily Lycopodioideae there appears to have been a complete loss of symbiosis in the *Lycopodiastrum-Pseudolycopodium-Austrolycopodium-Dendrolycopodium-Diphasium* clade (Field et al. 2016a). The low levels of colonization of lycophytes by symbiotic fungi and the evolution of non-symbiotic species suggest that these plants may have a low dependence on their mycorrhizal partners when mature (Rimington et al. 2015). On the other hand, the gametophytes of lycophytes are often subterranean and achlorophyllous and therefore fully dependent on their symbiotic fungi for nutrition (Schmid and Oberwinkler 1993).

### Identifying lycophyte fungal symbionts

The identity of the fungi that enter into symbiosis with lycophytes and the extent of these symbioses remain poorly resolved. While the available evidence indicates that only Mucoromycotina and Glomeromycotina colonize members of this lineage (Pressel et al. 2016), more work is needed to confirm this and to determine which symbionts dominate in nature (Lehnert et al. 2017). Symbiosis with Glomeromycotina has been reported more frequently than with Mucoromycotina (53 species vs. 6); however, most of these reports precede the discovery of Mucoromycotina-plant symbiosis and also lack molecular identification. Indeed, a recent molecular survey found a smaller difference in incidence of colonization between the two fungal lineages, albeit with Glomeromycotina also being the dominant type (Rimington et al. 2015). There has only been one report of colonization by Basidiomycota in lycophytes (Horn et al. 2013). However, because of a lack of electron microscopy evidence and of molecular methods suitable for detecting Mucoromycotina in this report, and as it contradicts all previous and subsequent reports (Table 1), its conclusion has been called into doubt. Reassessing the published images in Horn et al. (2013), Strullu-Derrien et al. (2014) proposed that the colonizing fungus more likely belongs to Mucoromycotina than Basidiomycota. Dark-septate endophytes (DSE) are Ascomycota fungi (Pressel et al. 2016) and so far have been recorded in ten lycophyte species from all three lycophyte families. However, there is no evidence that DSE may form mutualistic associations with lycophytes (Pressel et al. 2016). Thus, at present, only Glomeromycotina and Mucoromycotina can be considered mycorrhizal partners of this early-divergent vascular plant lineage.

## Conclusions

In concluding their seminal work, Wang and Qiu (2006) highlighted that ‘more basal land plants should be investigated, as they occupy an especially important position in our understanding of the origin of mycorrhizal symbiosis’. In the subsequent 13 years considerable effort has gone into addressing some of these gaps in knowledge so that the fungal symbiosis status of more than three times the number of early-diverging species reported in Wang and Qiu is now known. Nevertheless, further research is still required as to date only 6% of liverwort, 13% of hornwort and 5% of lycophyte species have been examined. Within liverworts, our survey highlights Jungermanniidae as the group in most need of further investigation. Lycophytes also require further investigation; it is likely that estimates of the occurrence of Mucoromycotina symbiosis in this lineage will increase with additional use of molecular methods.

Compiling this survey of fungal symbioses in early-diverging plants has highlighted the importance of both DNA sequencing and microscopy for determining the identity of plant fungal symbionts. Microscopy alone is not enough to identify fungi unless they display truly diagnostic characteristics; DNA sequencing allows us to determine fungal presence, but not whether this represents a symbiosis. Combining these two complementary methods is essential to fully understand the distribution and diversity of fungal symbiosis in plants, while physiological studies of resource exchange between partners are needed to assess whether the plant-fungus association is functionally mycorrhizal or mycorrhizal-like.

## Electronic supplementary material

ESM 1 (DOCX 20 kb)

ESM 2 (XLSX 14 kb)

## References

[CR1] Adams DG, Duggan PS (2008). Cyanobacteria-bryophyte symbioses. J Exp Bot.

[CR2] Bidartondo MI, Duckett JG (2010). Conservative ecological and evolutionary patterns in liverwort-fungal symbioses. Proc Royal Soc B Biol Sci.

[CR3] Bidartondo MI, Read DJ, Trappe JM, Merckx V, Ligrone R, Duckett JG (2011). The dawn of symbiosis between plants and fungi. Biol Lett.

[CR4] Brundrett MC (2009). Mycorrhizal associations and other means of nutrition of vascular plants: understanding the global diversity of host plants by resolving conflicting information and developing reliable means of diagnosis. Plant Soil.

[CR5] Brundrett MC, Tedersoo L (2018). Evolutionary history of mycorrhizal symbioses and global host plant diversity. New Phytol.

[CR6] Davis EC, Shaw AJ (2008). Biogeographic and phylogenetic patterns in diversity of liverwort-associated endophytes. Am J Bot.

[CR7] de Sousa F, Foster PG, Donoghue PCJ, Schneider H, Cox CJ (2019). Nuclear protein phylogenies support the monophyly of the three bryophyte groups (Bryophyta Schimp.). New Phytol.

[CR8] Desirò A, Duckett JG, Pressel S, Villarreal JC, Bidartondo MI (2013). Fungal symbioses in hornworts: a chequered history. Proc R Soc Lond B Biol Sci.

[CR9] Duckett JG, Ligrone R (2008). A cytological analysis of basidiomycetous endophytes in New Zealand Aneuraceae (simple thalloid liverworts, Metzgeriidae); confirmation of the derived status of *Verdoornia*. Can J Bot.

[CR10] Fehrer J, Réblová M, Bambasová V, Vohník M (2019). The root-symbiotic *Rhizoscyphus ericae* aggregate and *Hyaloscypha* (Leotiomycetes) are congeneric: phylogenetic and experimental evidence. Stud Mycol.

[CR11] Feldberg K, Vana J, Zhu RL, Heinrichs J (2010). The systematic position of *Pedinophyllum* (Marchantiophyta: Jungermanniales). Cryptogamie Bryol.

[CR12] Field KJ, Cameron DD, Leake JR, Tille S, Bidartondo MI, Beerling DJ (2012) Contrasting arbuscular mycorrhizal responses of vascular and non-vascular plants to a simulated Palaeozoic CO_2_ decline. Nat Commun 3:835 10.1038/ncomms1831 22588297

[CR13] Field KJ, Rimington WR, Bidartondo MI, Allinson KE, Beerling DJ, Cameron DD, Duckett JG, Leake JR, Pressel S (2015). First evidence of mutualism between ancient plant lineages (Haplomitriopsida liverworts) and Mucoromycotina fungi and its response to simulated Palaeozoic changes in atmospheric CO_2_. New Phytol.

[CR14] Field AR, Testo W, Bostock PD, Holtum JAM, Waycott M (2016a) Molecular phylogenetics and the morphology of the Lycopodiaceae subfamily Huperzioideae supports three genera: *Huperzia*, *Phlegmariurus* and *Phylloglossum*. Mol Phylogenetics Evol 94:635–657 10.1016/j.ympev.2015.09.024 26493224

[CR15] Field KJ, Rimington WR, Bidartondo MI, Allinson KE, Beerling DJ, Cameron DD, Duckett JG, Leake JR, Pressel S (2016). Functional analysis of liverworts in dual symbiosis with Glomeromycota and Mucoromycotina fungi under a simulated Palaeozoic CO_2_ decline. ISME J.

[CR16] Field KJ, Bidartondo MI, Rimington WR, Hoysted GA, Beerling DJ, Cameron DD, Duckett JG, Leake JR, Pressel S (2019). Functional complementarity of ancient plant–fungal mutualisms: contrasting nitrogen, phosphorus and carbon exchanges between Mucoromycotina and Glomeromycotina fungal symbionts of liverworts. New Phytol.

[CR17] Flores JR, Catalano SA, Muñoz J, Suárez GM (2017). Combined phylogenetic analysis of the subclass Marchantiidae (Marchantiophyta): towards a robustly diagnosed classification. Cladistics.

[CR18] Forrest LL, Davis EC, Long DG, Crandall-Stotler BJ, Clark A, Hollingsworth ML (2006). Unraveling the evolutionary history of the liverworts (Marchantiophyta): multiple taxa, genomes and analyses. Bryologist.

[CR19] Harley JL, Harley EL (1987). A check-list of mycorrhiza in the British flora. New Phytol.

[CR20] Hassler M, Schmitt B (2018) Checklist of ferns and lycophytes of the world version 7.4. Available at: http://worldplants.webarchiv.kit.edu/ferns/index.php

[CR21] Hibbett DS, Binder M, Bischoff JF, Blackwell M, Cannon PF, Eriksson OE, Huhndorf S, James T, Kirk PM, Lucking R (2007). A higher-level phylogenetic classification of the fungi. Mycol Res.

[CR22] Horn K, Franke T, Unterseher M, Schnittler M, Beenken L (2013). Morphological and molecular analyses of fungal endophytes of achlorophyllous gametophytes of *Diphasiastrum alpinum* (Lycopodiaceae). Am J Bot.

[CR23] Hoysted GA, Jacob AS, Kowal J, Giesemann P, Bidartondo MI, Duckett JG, Gebauer G, Rimington WR, Schornack S, Pressel S, Field KJ (2019). Mucoromycotina fine root endophyte fungi form nutritional mutualisms with vascular plants. Plant Physiol.

[CR24] Kenrick P, Crane PR (1997). The origin and early evolution of plants on land. Nature.

[CR25] Kostka JE, Weston DJ, Glass JB, Lilleskov EA, Shaw J, Turetsky MR (2016). The *Sphagnum* microbiome: new insights from an ancient plant lineage. New Phytol.

[CR26] Kowal J, Pressel S, Duckett JG, Bidartondo MI, Field KJ (2018). From rhizoids to roots? Experimental evidence of mutualism between liverworts and ascomycete fungi. Ann Bot.

[CR27] Lehnert M, Krug M, Kessler M (2017). A review of symbiotic fungal endophytes in lycophytes and ferns – a global phylogenetic and ecological perspective. Symbiosis.

[CR28] Ligrone R, Carafa A, Lumini E, Bianciotto V, Bonfante P, Duckett JG (2007). Glomeromycotean associations in liverworts: a molecular cellular and taxonomic analysis. Am J Bot.

[CR29] Masuzaki H, Shimamura M, Furuki T, Tsubota H, Yamaguchi T, Majid HMA, Deguchi H (2010). Systematic position of the enigmatic liverwort *Mizutania* (Mizutaniaceae, Marchantiophyta) inferred from molecular phylogenetic analyses. Taxon.

[CR30] Orchard S, Hilton S, Bending GD, Dickie IA, Standish RJ, Gleeson DB, Jeffery RP, Powell JR, Walker C, Bass D (2017). Fine endophytes (*Glomus tenue*) are related to Mucoromycotina, not Glomeromycota. New Phytol.

[CR31] Paton JA (1999). The liverwort flora of the British Isles.

[CR32] Patzak SDF, Renner MAM, Schäfer-Verwimp A, Feldberg K, Heslewood MM, Peralta DF, de Souza AM, Schneider H, Heinrichs J (2016). A phylogeny of Lophocoleaceae-Plagiochilaceae-Brevianthaceae and a revised classification of Plagiochilaceae. Org Divers Evol.

[CR33] Pimm SL, Raven PH (2017). The fate of the world’s plants. Trends Ecol Evol.

[CR34] Pocock K, Duckett JG (1985). On the occurrence of branched and swollen rhizoids in British hepatics: their relationships with the substratum and associations with fungi. New Phytol.

[CR35] PPG1 (2016). A community derived classification for extant lycophytes and ferns. J Sys Evol.

[CR36] Pressel S, Bidartondo MI, Ligrone R, Duckett JG (2010). Fungal symbioses in bryophytes: new insights in the twenty first century. Phytotaxa.

[CR37] Pressel S, Bidartondo MI, Field KJ, Rimington WR, Duckett JG (2016). Pteridophyte fungal associations: current knowledge and future perspectives. J Sys Evol.

[CR38] Puttick MN, Morris JL, Williams TA, Cox CJ, Edwards D, Kenrick P, Pressel S, Wellman CH, Schneider H, Pisani D, Donoghue PCJ (2018). The interrelationships of land plants and the nature of the ancestral embryophyte. Curr Biol.

[CR39] Rabeau L, Gradstein SR, Dubuisson J, Nebel M, Quandt D, Reeb C (2017). New insights into the phylogeny and relationships within the worldwide genus *Riccardia* (Aneuraceae, Marchantiophytina). Eur J Taxon.

[CR40] Remy W, Taylor TN, Hass H, Kerp H (1994). Four hundred-million-year-old vesicular-arbuscular mycorrhizae. PNAS.

[CR41] Renzaglia KS, Schuette S, Duff RJ, Ligrone R, Shaw AJ, Mishler BD, Duckett JG (2007). Bryophyte phylogeny: advancing the molecular and morphological frontiers. Bryologist.

[CR42] Rikkinen J, Virtanen V (2008). Genetic diversity in cyanobacterial symbionts of thalloid bryophytes. J Exp Bot.

[CR43] Rimington WR, Pressel S, Duckett JG, Bidartondo MI (2015). Fungal associations of basal vascular plants: reopening a closed book?. New Phytol.

[CR44] Rimington WR, Pressel S, Duckett JG, Field KJ, Read DJ, Bidartondo MI (2018). Ancient plants with ancient fungi: liverworts associate with early-diverging arbuscular mycorrhizal fungi. Proc R Soc Lond B Biol Sci.

[CR45] Rimington WR, Pressel S, Duckett JG, Field KJ, Bidartondo MI (2019). Evolution and networks in ancient and widespread symbioses between Mucoromycotina and liverworts. Mycorrhiza.

[CR46] Schmid E, Oberwinkler F (1993). Mycorrhiza-like interaction between the achlorophyllous gametophyte of *Lycopodium clavatum* L. and its fungal endophyte studied by light and electron-microscopy. New Phytol.

[CR47] Schuster RM (1969). The Hepaticae and Anthocerotae of North America, Volume 2.

[CR48] Schuster RM (1980). The Hepaticae and Anthocerotae of North America, Volume 4.

[CR49] Shaw B, Crandall-Stotler B, Váňa J, Stotler RE, von Konrat M, Engel JJ, Davis EC, Long DG, Sova P, Shaw AJ (2015). Phylogenetic relationships and morphological evolution in a major clade of leafy liverworts (phylum Marchantiophyta, order Jungermanniales): suborder Jungermanniineae. Syst Bot.

[CR50] Smith SE, Read DJ (1997). Mycorrhizal symbiosis.

[CR51] Smith SE, Read DJ (2008). Mycorrhizal symbiosis.

[CR52] Söderström L, Hagborg A, von Konrat M, Bartholomew-Began S, Bell D, Briscoe L, Brown E, Cargill DC, Costa DP, Crandall-Stotler BJ et al (2016) World checklist of hornworts and liverworts. Phytokeys 59:1–828 10.3897/phytokeys.59.6261 PMC4758082 26929706

[CR53] Stahl M (1949). Die Mycorrhiza der Lebermoose mit besonderer Berucksichtigung der thallosen formen. Planta.

[CR54] Stotler RE, Crandall-Stotler B (2017). A synopsis of the liverwort flora of North America north of Mexico. Ann Mo Bot Gard.

[CR55] Strullu-Derrien C, Kenrick P, Pressel S, Duckett JG, Rioult J-P, Strullu D-G (2014). Fungal associations in *Horneophyton ligneri* from the Rhynie Chert (c. 407 million year old) closely resemble those in extant lower land plants: novel insights into ancestral plant–fungus symbioses. New Phytol.

[CR56] Upson R, Read DJ, Newsham KK (2007). Widespread association between the ericoid mycorrhizal fungus *Rhizoscyphus ericae* and a leafy liverwort in the maritime and sub-Antarctic. New Phytol.

[CR57] Villarreal JC, Renner SS (2013). Correlates of monoicy and dioicy in hornworts, the apparent sister group to vascular plants. BMC Evol Biol.

[CR58] Villarreal JC, Renzaglia KS (2006). Structure and development of *Nostoc* strands in *Leiosporoceros dussii* (Anthocerotophyta): a novel symbiosis in land plants. Am J Bot.

[CR59] Wang B, Qiu YL (2006). Phylogenetic distribution and evolution of mycorrhizas in land plants. Mycorrhiza.

[CR60] Warshan D, Espinoza JL, Stuart RK, Richter RA, Kim SY, Shapiro N, Woyke T, Kyrpides NC, Barry K, Singan V (2017). Feathermoss and epiphytic *Nostoc* cooperate differently: expanding the spectrum of plant-cyanobacteria symbiosis. The ISME J.

[CR61] Yamamoto K, Shimamura M, Degawa Y, Yamada A (2019). Dual colonization of Mucoromycotina and Glomeromycotina fungi in the basal liverwort, *Haplomirium mnioides* (Haplomitriopsida). J Plant Res.

