# Supplementary material for: The distribution and evolution of fungal symbioses in ancient lineages of land plants
Source: Mycorrhiza. 2020 Mar 4;30(1):23–49. doi: 10.1007/s00572-020-00938-y (PMC7062687; doi:10.1007/s00572-020-00938-y)
Supplement: Supplementary file 1 — (DOCX 20 kb) [file 572_2020_938_MOESM1_ESM.docx]

**Supplementary material**

**The distribution and evolution of fungal symbioses in ancient lineages of land plants.** *Mycorrhiza*

William R. Rimington^1,2,3^, Jeffrey G. Duckett^2^, Katie J. Field^4^, Martin I. Bidartondo^1,3^ and Silvia Pressel*^2^

^1^Department of Life Sciences. Imperial College London. London, SW7 2AZ, UK.

^2^Department of Life Sciences, Algae, Fungi and Plants Division. Natural History Museum, London. London, SW7 5BD, UK.

^3^Comparative Plant and Fungal Biology, Royal Botanic Gardens, Kew. Richmond, TW9 3DS, UK.

^4^Centre for Plant Sciences, Faculty of Biological Sciences. University of Leeds. Leeds, LS2 9JT, UK.

*Corresponding author: s.pressel@nhm.ac.uk

**Contents**

Tables S1, S2

**Table S1. Species numbers used for estimating fungal symbiosis occurrence rates.** Species numbers for liverwort families and hornwort genera were acquired from Soderstrom et al. (2016). Lycophyte species numbers were acquired from Hassler and Schmitt (2018).

Table included as separate excel file.

**References**

Hassler M, Schmitt B (2018) Checklist of Ferns and Lycophytes of the World version 7.4. Available at: http://worldplants.webarchiv.kit.edu/ferns/index.php.

Soderstrom L, Hagborg A, von Konrat M, Bartholomew-Began S, Bell D, Briscoe L, Brown E, Cargill DC, Costa DP, Crandall-Stotler BJ, et al. (2016) World checklist of hornworts and liverworts. Phytokeys 59:1-828

**Table S2.** Liverwort species for which information on their fungal symbiosis status was obtained from either the Flora of Paton (1999) or from our own unpublished microscopy observations. Paton JA (1999) The Liverwort Flora of the British Isles. Leiden: Brill.

| **Species** | **Year of collection; locality** | **Habitat** | **Personal Observation (PO)/reference** |
| --- | --- | --- | --- |
| *Moerckia flotoviana* | 2013; Anglesey, Wales | Dune slacks | PO |
| *Adelanthus lindenbergianus* | 2013; Sani Pass, Lesotho | South-facing cliff | PO |
| *Pseudomarsupidium (Adelanthus) decipiens* | 2013; Merioneth, Wales | Mossy bank | PO |
| *Anastrophyllum alpinum* | 2010; West Ross, Scotland | Turfy alpine ledge | PO |
| *Anastrophyllum joergensenii* | 2010; West Ross, Scotland | Turfy alpine ledge | PO |
| *Orthocaulis (Barbilophozia)* *atlanticus* | 2013; Merioneth, Wales | Boulder scree | PO |
| *Schljakovia (Barbilophozia) kunzeana* |  |  | Paton, 1999 |
| *Cephalozia ambigua* | 2010; East Ross, Scotland | Late snow patch | PO |
| *Odontoschisma macounii* | 2010; West Ross, Scotland | Thin soil on cliff | PO |
| *Cephaloziella turneri* | 2011; Cornwall, England | Damp bank | PO |
| *Gymnomitrion corallioides* | 2012; Mount Washington, New Hampshire, USA | Bare soil | PO |
| *Hygrobiella laxifolia* | 2013; Caernarvonshire, Wales | Wet rocks in stream | PO |
| *Mesoptychia (Leiocolea) badensis* | 2013; Anglesey, Wales | Dune slacks | PO |
| *Telaranea europaea* | 2013; Caernarvonshire, Wales | Peaty bank | PO |
| *Tricholepidozia* *(Telaranea) tetradactyla* | 2010; Surrey, England | Woodland bank | PO |
| *Lophocolea bispinosa* | 2011; Surrey, England | Woodland floor | PO |
| *Lophocolea brookwoodiana* | 2011; Surrey, England | Woodland floor | PO |
| *Lophocolea fragrans* | 2013; Merioneth, Wales | Woodland bank | PO |
| *Lophocolea semiteres* | 2011; Surrey, England | Woodland floor | PO |
| *Trilophozia (Tritomaria) quinquedentata* |  |  | Paton, 1999 |
| *Plagiochila bifaria* | 2013; Merioneth, Wales | Mossy boulders | PO |
| *Plagiochila britannica* | 2013; Merioneth, Wales | Mossy boulders | PO |
| *Diplophyllum obtusatum* | 2010; Tennessee, USA | Woodland bank | PO |
| *Scapania paludicola* | 2010; East Ross, Scotland | Alpine flush | PO |
| *Scapania subalpina* | 2013; Caernarvonshire, Wales | Turfy ledge | PO |
